# Sex Differences in the Patterns and Predictors of Cognitive Function in HIV

**DOI:** 10.3389/fneur.2020.551921

**Published:** 2020-11-23

**Authors:** Leah H. Rubin, Erin E. Sundermann, Raha Dastgheyb, Alison S. Buchholz, Elizabeth Pasipanodya, Robert K. Heaton, Igor Grant, Ronald Ellis, David J. Moore

**Affiliations:** ^1^Department of Neurology, Johns Hopkins University, Baltimore, MD, United States; ^2^Department of Psychiatry, Johns Hopkins University, Baltimore, MD, United States; ^3^Department of Epidemiology, Johns Hopkins University, Baltimore, MD, United States; ^4^Department of Psychiatry, University of California, San Diego, La Jolla, CA, United States; ^5^Rehabilitation Research Center, Santa Clara Valley Medical Center, San Jose, CA, United States; ^6^Department of Neurosciences, University of California, San Diego, La Jolla, CA, United States

**Keywords:** HIV, sex differences, cognition, women, neuropsychology, cognitive profiles

## Abstract

Despite advancements in antiretroviral therapy, mild cognitive deficits persist in nearly half of people with HIV (PWH). The profile of impairment in HIV is highly variable with deficits observed in a range of cognitive domains. Despite evidence of greater cognitive impairment among women with HIV (WWH) vs. men with HIV (MWH), it is unclear how MWH and WWH differ in the type of cognitive impairment and in risk factors associated with cognitive impairment profiles. In a large and well-characterized sample of PWH, we used machine learning to identify profiles of cognitive functioning and their associated factors overall and within sex. Participants included 1,666 PWH (201 WWH; 1,465 MMH) from the HIV Neurobehavioral Research Program who completed a neuropsychological test battery at their baseline visits. Using demographically-adjusted T-scores from 13 test outcomes assessing motor skills, executive functioning, attention/working memory, episodic learning and memory, verbal fluency, and processing speed, we used Kohonen self-organizing maps to identify patterns of high-dimensional data by mapping participants to similar nodes based on T-scores (MCLUST R package). Random forest models were used to determine how sociodemographic (e.g., age, education), clinical (e.g., depressive symptoms, substance use disorder), and biological (e.g., HIV disease characteristics) factors differentially related to membership within a cognitive profile. All analyses were repeated within sex. Three cognitive profiles were identified overall and within each sex. Overall and within MWH, there were unimpaired and global weakness profiles. The third profile in the total sample demonstrated relatively weak auditory attention whereas in MWH showed relative strengths in attention and processing speed. Conversely, there was no unimpaired profile among WWH. Rather, WWH demonstrated separate profiles reflecting weakness in motor skills, a relative weakness in learning and delayed recall, and global weaknesses with spared recognition memory. Despite different cognitive profiles by sex, the most discriminative factors were similar between men and women and included reading level (cognitive reserve), current and nadir CD4 count, plasma HIV viral load, duration of HIV disease, age, depressive symptoms, and race/ethnicity. Findings fill a knowledge gap concerning sex differences in cognitive impairment in PWH and inform personalized risk reduction and therapeutic strategies.

## Introduction

The Human Immunodeficiency Virus (HIV) enters the central nervous system (CNS) within days of initial infection (1), in many cases leading to neurological, cognitive, and behavioral complications. Cognitive deficits are a common feature of HIV/AIDS. While the incidence of HIV-associated dementia has considerably decreased in the era of modern ART suppressing viral replication, mild cognitive deficits with no change in everyday function persist in 24% [95% confidence interval (CI) = 20.3–26.8] of people with HIV (PWH) and mild cognitive deficits with mildly decreased everyday function persist in about 13.3% (95% CI = 10.6–16.3) of PWH (2). Although executive function and memory deficits are most common in PWH in the post-ART era, the characterization of cognitive impairment in HIV is highly variable with deficits observed in a range of cognitive domains (3). Previous studies using statistical clustering techniques have identified differing profiles of cognitive function among PWH with some profiles resembling global impairment across domains while other profiles resemble more domain-specific impairment, particularly in the domains of episodic memory and executive function (4–7). Similarly, there is also substantial variability in the risk factors associated with cognitive deficits among PWH that range from biological (e.g., CD4^+^ T-cell count, HIV viral load, comorbid health conditions), demographic (e.g., age, sex, race/ethnicity) to psychosocial factors (e.g., low education, depression, substance use/dependence). The persistence of cognitive impairment in the era of modern ART among PWH and the variability in the profiles and risk factors associated with cognitive impairment suggests that non-HIV factors associated with aging, comorbid conditions (e.g., cardiovascular disease) and psychosocial risk factors (e.g., poverty, poor education) likely contribute to cognitive impairment given the high prevalence of these factors among PWH (8, 9). With this in mind, we propose looking beyond the construct of HIV-associated neurocognitive disorders (HAND) to identify the underlying pathophysiology linked to cognitive impairment as HAND requires other comorbidities to be ruled out as primary contributing factors.

Biological sex is an important determinant of cognitive impairment among PWH. In a recent literature review of sex differences in cognitive impairment among PWH (10), seven cross-sectional (11–17) and one longitudinal analysis (18) identified sex differences on global measures of cognitive impairment among PWH. Additionally, six cross-sectional (13–15, 17, 19, 20) and one longitudinal analysis (21) also reported sex differences in domain-specific cognitive performance. The strongest available evidence of adequately-powered studies indicates that WWH show greater deficits than MWH in the domains of learning and memory followed by speed of information processing and motor functioning, with inconsistent findings in executive functioning (17, 21).

The greater vulnerability of WWH to cognitive impairment may reflect sociodemographic differences between men and women with HIV. WWH tend to have a higher prevalence of psychosocial risk factors including poverty, low literacy levels, low educational attainment, substance abuse, poor mental health, and barriers to health care services (10, 22) as compared to MWH. These psychosocial risk factors may have biological effects on the brain that lead to reduced cognitive reserve among WWH (23, 24) as evidenced by findings of greater susceptibility of cognitive function to the effects of mental health factors (e.g., depression) among WWH vs. MWH (25). Additionally, biological factors such as sex steroid hormones (e.g., estrogen, testosterone) and female-specific hormonal milieus (e.g., pregnancy, menstrual cycle, menopause transition) may contribute to sex differences in cognitive test performance in PWH. However, it remains unclear how MWH and WWH may differ in the patterns of cognitive impairment and risk factors associated with these patterns of cognitive impairment. Previous reports of impairment profiles among PWH have identified them in combined samples of men and women (4–7), masking possible sex-specific patterns of cognitive impairment among PWH. Furthermore, although a number of studies reported sex differences in the presence and pattern of cognitive impairment (14, 16, 17) and greater cognitive decline compared to MWH (18), only one study (17) was adequately powered to address meaningful sex difference in global cognitive function (10). A well-powered examination of the patterns and determinants of cognitive impairment by sex, that also controls for other demographic differences between WWH and MWH (e.g., age, education, race/ethnicity), can help to clarify the contribution of sex to heterogeneity in cognitive impairment among PWH. Such an examination could also clarify the related psychosocial vs. biological factors and, thereby, optimize risk assessments and intervention strategies in both sexes.

Leveraging comprehensive neuropsychological (NP) data from the large-scale cohort of the HIV Neurobehavioral Research Program (HNRP) at the University of California-San Diego, we used novel machine learning methods to identify differing profiles of cognitive function in PWH and to evaluate how these profiles differ between women and men in sex-stratified analyses. Rather, than using traditional cognitive domain scores, we used each of the NP test outcomes given that prior studies indicate that the correlation of NP test scores does not map to traditional domain scores in PWH. Furthermore, we determined how sociodemographic (e.g., age, education, race/ethnicity), clinical (e.g., functional status, depression, substance use disorders) and biological (e.g., measures of HIV disease severity, ART use, cardiovascular comorbid conditions, Hepatitis C co-infection) factors related to cognitive profiles within women and men. Based on previous studies among PWH (4–6), we hypothesized that the machine learning approach would identify distinct subgroups of individuals with normal cognitive function, global cognitive impairment, and domain-specific cognitive impairment. We further hypothesized that groups with domain-specific cognitive impairment would differ by sex, with WWH showing more consistent memory and processing speed impairment than MWH. Finally, we expected that similar sociodemographic/clinical/biological determinants would distinguish cognitive profiles (e.g., age, education, race, HIV viral load) among WWH and MWH; however, in line with previous research (25), we expected that depressive symptoms would be more strongly associated with cognitive impairment profiles among WWH than MWH.

## Materials and Methods

### Participants

Participants included 1,666 PWH (201 WWH; 1,465 MWH) enrolled in various NIH-funded research studies at the University of California, San Diego's HNRP, https://hnrp.hivresearch.ucsd.edu/. Study assessment details have been published elsewhere (3). The UCSD Institutional Review Board approved the studies. Participants provided written informed consent and were compensated for their participation. Exclusion criteria for the parent studies included history of non-HIV-related neurological, medical, or psychiatric disorders that affect brain functioning (e.g., seizure, stroke, psychosis), learning disabilities, and a first language that was not English. Inclusion in the current study required completion of neuropsychological and neuromedical evaluations at the baseline study visit. Exclusion criteria for the current study included a positive urine toxicology test for illicit drugs (excluding marijuana) or Breathalyzer test for alcohol on the day of clinic visit on the day of study visit.

### NP Test Evaluation

NP test performance was assessed through a comprehensive, standardized, battery of tests that measure seven domains of cognition, including complex motor skills, executive function, attention/working memory, episodic learning, episodic memory (delayed recall and recognition), verbal fluency, and information processing speed. Motor skills were assessed by the Grooved Pegboard (GPEG) Dominant and Non-dominant Hand tests (26). Executive functioning was assessed by the Trail Making Test (TMT)-Part B (27) and the Stroop Color and Word Test interference score (28). Attention/working memory was assessed by the Paced Auditory Serial Addition Task (PASAT-50) (29, 30). Episodic learning was assessed by the Total Learning scores of the Hopkins Verbal Learning Test-Revised (HVLT-R) (31) and the Brief Visuospatial Memory Test-Revised (BVMT-R) (32). Episodic memory was assessed by the Delayed Recall and Recognition scores of the HVLT-R and BVMT-R. Verbal Fluency was assessed by the “FAS” Letter Fluency test (33). Information processing speed was assessed by the WAIS-III Digit Symbol Test (34), the TMT-Part A, and the Stroop Color and Word Test color naming score. Raw test scores were transformed into age-, education-, sex-, and race/ethnicity-adjusted T-scores based on normative samples of HIV-uninfected persons (35, 36). The use of demographically-adjusted T-scores are intended to control for these demographic effects as they occur in the general population.

### Factors Associated With NP Profiles

We examined sociodemographic, clinical, and biological factors associated with cognitive impairment in the literature and available with enough participants to be adequately powered in analyses. Sociodemographic factors included age, years of education, and race/ethnicity. Although these factors were used to create the T-scores, there can still be remaining demographic associations with cognition within clinical populations such as PWH. For example, there is considerable interest in the possibility of abnormal cognitive aging PWH; also, in general, older PWH tend to have had their infections longer, may have had longer periods without benefit of suppressive ART, and more history of worse immunosuppression. Clinical factors included functional status as indicated by the number of daily activities with decreased independence from the Instrumental Activities of Daily Living questionnaire (IADL) from the modified version of the Lawton and Brody Activities of Daily Living Questionnaire (37), reading level (a proxy for cognitive reserve) based on the Wide Range Achievement Test-4 Reading subtest (WRAT-4 Reading) (38), self-reported depressive symptoms on the Beck Depression Inventory versions I (BDI-I) or II (BDI-II) (39), and diagnosis of lifetime and current major depressive disorder (MDD) as well as lifetime alcohol, cannabis, or other (i.e., amphetamine, cocaine, hallucinogen, inhalant, sedative, opioid, and PCP) substance use disorder based on the Composite International Diagnostic Interview using DSM–IV criteria (CIDI version 2.1) (40). Biological factors included HIV disease variables such as current CD4^+^ T-cell count, lowest CD4^+^ T-cell count ever recorded (nadir CD4), plasma HIV viral load, estimated duration of HIV disease, current use of ART, current use of anticholinergic-based medications (e.g., urinary incontinence and chronic obstructive pulmonary disease medications), Hepatitis C co-infection, and the cardiovascular comorbid conditions of hypertension, hyperlipidemia, and diabetes.

### Statistical Analyses

All 13 NP tests were used to find groups of similar cognitive profiles within each participant subset (MWH, WWH) and in the total sample using a pipeline that consisted of dimension reduction with Kohonen self-organizing maps (SOM) followed by clustering to identify profiles based on those reduced dimensions. SOM was implemented using the Kohonen package in R (41). SOM is an unsupervised machine learning technique used to identify patterns in high-dimensional data (numerous variables) by producing a two-dimensional representation consisting of multiple nodes where each node is a group of one or more individuals with similar cognitive profiles and the location of the nodes within the 2-D representation is also a metric of similarity. Unlike probabilistic models, each individual can only be assigned to one node. The SOM grid consisted of a 10 × 10 hexagonal grid of nodes and the number of clusters for the final profiling was selected by looping over models created from 3 to 20 clusters and selecting the number that had the best fit based on entropy. Similar nodes were then clustered (grouped together) using the MClust package (42). MClust is an R Software package used for model-based clustering using finite normal mixture modeling that provides functions for parameter estimation via the Expectation-Maximization algorithm with an assortment of covariance structures which vary in distribution (spherical, diagonal, or ellipsoidal), volumes (equal or variable), shape (equal of variable), and orientation (equal or variable, only for ellipsoidal distribution). This program identifies the best model based on entropy (a model fit statistic). Once the clustering of the nodes was completed, cluster profiles were assigned to the individuals associated with that node. By using SOM and MClust in sequence, we were able to achieve fine-tuned clustering based on patterns of performance in cognitive testing.

Factors predicting profile membership between each impaired and unimpaired profile in the overall sample and within each group (MWH, WWH) were explored by creating a predictive Random Forest (RF) model using the Caret (43) package in R and then extracting variable importance (44). RF is an ensemble machine learning model based on classification trees that results in powerful prediction models based on non-linear combinations of subsets of input variables. Prior to model creation, the Synthetic Minority Over-sampling Technique (SMOTE) with the DMwR (45) package was used to control for bias due to any imbalance in the number of cases. RF models were created using internal validation using a 10-fold resampling method repeated 5 times. Pre-processing before RF creation involved removing variables as predictors if they had low variance or if they had >50% missing data. Any missing data in the remaining variables was imputed using the Multivariate Imputation by Chained Equations (46) (MICE) package in R using random forest imputations. ROC confidence intervals were calculated using the pROC package in R with 2,000 stratified bootstrap replicates (95% CI). Variable importance of all variables included in the RF models was used as the outcome metric of the predictive power of each variable. Variable importance is a scaled number [0–100] that indicates how important that variable is to the final predicted outcome in that model. For each tree in the RF model, the out-of-bag portion of the data is recorded and repeated after permuting each predictor variable. The difference between the accuracy with and without each variable is averaged over all trees and then normalized by the standard error. For visualization, all variables were plotted by relative variable importance, and attention was given to the top 10 variables in each profile. Variable importance indicates how much that variable contributes to overall prediction accuracy, but as RF is non-linear model it does not indicate directionality.

While the analysis pipeline and packages used along with the parameter inputs are stated above, we have added our code into a Supplementary Material to facilitate rigor and reproducibility.

## Results

### Participants

Table 1 provides sociodemographic, behavioral, and clinical factors for 1,666 PWH (1,465 men; 201 women). On average, participants were 41.8 years of age [standard deviation (SD) = 9.8] with 13.3 years of education (SD = 2.7). Fifty-eight percent were White and 18% Black. Mental health comorbidities were common. Forty-eight percent had a lifetime, and 19% had a current, diagnosis of MDD. With respect to HIV-related clinical characteristics, 60% were on combination ART and 42% were virally suppressed. Compared to MWH, WWH were less educated, had lower WRAT-4 scores, were less likely to be white, and had a shorter duration of HIV disease (*P*'s < 0.05). Additionally, WWH reported more IADL dependence and were more likely to have HCV co-infection compared to MWH (*P*'s < 0.05).

**Table 1:** Demographic, behavioral, and clinical characteristics in the total sample of people with HIV and by sex.

		**Sex**		
	**Total**	**Men**	**Women**	
	**(*N* = 1,666)**	**(*n* = 1,465)**	**(*n* = 201)**	
	***n* (%)**	***n* (%)**	***n* (%)**	***P*-value**
Age, M (SD)	41.7 (9.8)	41.9 (9.8)	40.7 (9.6)	0.09
Years of education, M (SD)	13.3 (2.7)	13.5 (2.7)	12.0 (2.5)	<0.001
WRAT-4, M (SD)	99.1 (13.5)	99.8 (13.3)	93.5 (13.2)	<0.001
Race				<0.001
White	971 (58)	890 (61)	81 (40)	
Black	314 (19)	259 (18)	55 (28)	
Hispanic	299 (18)	246 (17)	53 (26)	
Other	82 (5)	70 (4)	12 (6)	
IADL dependence	2.5 (2.9)	2.4 (2.8)	2.9 (3.1)	0.03
BDI-II	13.5 (10.4)	13.5 (10.4)	13.5 (9.9)	0.99
DSM-IV (CIDI) diagnoses				
MDD				
Current	224 (19)	203 (19)	21 (18)	0.94
Lifetime	572 (48)	510 (48)	572 (48)	0.24
Alcohol (current or lifetime^*^)	72 (6)	66 (6)	6 (5)	0.84
Cannabis (current or lifetime^*^)	63 (5)	60 (6)	3 (3)	0.25
Substance use (current or lifetime^*^)	691 (73)	627 (73)	64 (71)	0.74
Anticholinergic medications	422 (25)	367 (25)	55 (27)	0.53
Hypertension	334 (21)	298 (21)	36 (18)	37
Hyperlipidemia	219 (14)	193 (14)	26 (13)	0.9
Diabetes	82 (5)	70 (5)	12 (6)	0.64
HCV	317 (20)	259 (18)	58 (29)	<0.001
Log plasma viral load, M (SD)	3.1 (1.3)	3.1 (1.3)	3.1 (1.2)	0.83
CD4 count, M (SD)				
Current	429.0 (296.9)	426.9 (295.8)	443.7 (305.3)	0.46
Nadir	229.9 (219.9)	250.4 (231.8)	232.4 (221.4)	0.21
Duration of HIV disease, M (SD)	9.1 (7.8)	9.3 (8.0)	7.0 (5.6)	0.005
AIDS diagnosis	969 (58)	854 (59)	115 (57)	0.07
On ART	966 (60)	857 (60)	109 (56)	0.3

*M, mean; SD, standard deviation; ART, antiretroviral therapy; WRAT-4, Wide Range Achievement Test; IADL, Instrumental Activities of Daily Living; BDI, Beck depression inventory; CIDI, Composite International Diagnostic Interview; HCV, Hepatitis C*.

* *Current or Lifetime Use disorder (abuse or dependence)*.

Table 2 provides NP test performance for the total sample and by sex. In the total sample, average performance on BVMT-R delayed recall, HVLT-R (total learning, delayed recall, recognition), GPEG-non-dominant, and PASAT had T-scores <45 or 0.5 standard deviations from the general population mean (T-score of 50). WWH performed worse than MWH on BVMT-R total learning on average (*P* = 0.01). However, WWH performed better on the recognition measures of the BVMT-R and HVLT-R (*P*'s < 0.01). This was also the case examining percent impairment using a T-score cutoff of 40 (*P*'s < 0.001).

**Table 2:** Neuropsychological test performance in the total sample of people with HIV and by sex.

		**Sex**		
	**Total**	**Men**	**Women**	
	**(*N* = 1,666)**	**(*n* = 1,465)**	**(*n* = 201)**	***P*-value**
**T-scores**	**M (SD)**	**M (SD)**	**M (SD)**	
**BVMT-R**				
Total learning	45.4 (10.0)	45.6 (10.1)	43.84 (9.6)	0.01
Delayed recall	42.2 (11.4)	45.2 (11.5)	45.6 (11.1)	0.6
Recognition	45.8 (13.7)	45.5 (13.7)	48.3 (13.1)	0.006
**HVLT-R**				
Total learning	42.6 (11.6)	42.5 (11.7)	43.0 (11.1)	0.56
Delayed recall	43.2 (11.7)	43.3 (11.8)	42.6 (10.8)	0.41
Recognition	44.4 (13.8)	43.6 (13.6)	50.5 (13.3)	<0.001
**Grooved pegboard**				
Dominant	45.4 (11.9)	45.4 (11.8)	45.4 (12.5)	0.98
Non-dominant	44.7 (11.3)	44.7 (11.3)	44.4 (11.6)	0.7
**Trail making test**				
Part A	48.3 (11.9)	48.2 (11.9)	49.1 (11.7)	0.31
Part B	46.3 (11.7)	46.1 (11.7)	47.4 (11.4)	0.14
Letter fluency	47.0 (11.5)	47.0 (11.2)	46.9 (13.2)	0.96
PASAT 50	44.7 (11.6)	44.7 (11.6)	44.9 (11.8)	0.79
Digit symbol test	47.2 (11.2)	47.3 (11.3)	46.9 (10.7)	0.67
**PERCENT IMPAIRMENT (40 CUTPOINT)**	***N*** **(%)**	***n*** **(%)**	***n*** **(%)**	
**BVMT-R**				
Total learning	429 (29)	499 (30)	70 (34)	0.13
Delayed recall	483 (33)	546 (33)	63 (31)	0.70
Recognition	434 (30)	487 (29)	53 (26)	0.38
**HVLT-R**				
Total learning	595 (41)	670 (40)	75 (37)	0.41
Delayed recall	564 (38)	641 (38)	77 (38)	1.00
Recognition	476 (33)	510 (31)	34 (17)	<0.001
**Grooved pegboard**				
Dominant	470 (32)	536 (32)	66 (33)	0.89
Non-dominant	461 (31)	523 (31)	62 (31)	0.92
**Trail making test**				
Part A	297 (20)	341 (20)	44 (22)	0.66
Part B	410 (28)	457 (27)	47 (23)	0.20
Letter fluency	342 (23)	396 (24)	54 (27)	0.31
PASAT 50	508 (35)	580 (35)	72 (36)	0.81
Digit symbol test	378 (26)	430 (26)	52 (26)	1.00

*M, mean; SD, standard deviation; BVMT-R, Benton Visual Retention Test-Revised; HVLT-R, Hopkins Verbal Learning Test-Revised; PASAT, Paced Auditory Serial Addition Test*.

### Identification of Cognitive Profiles in the Total Sample

Profiles where the mean T-score on all cognitive outcomes was >45 and <55 were considered an “unimpaired average” profile. To describe the profiles, tests where the average T-scores of all participants in that cluster were <45 were considered weaknesses, and those where the average was <40 were considered impaired. An average >55 was considered a relative strength in the context of other domains being in the average range (>45 and <55).

**Figure 1:**
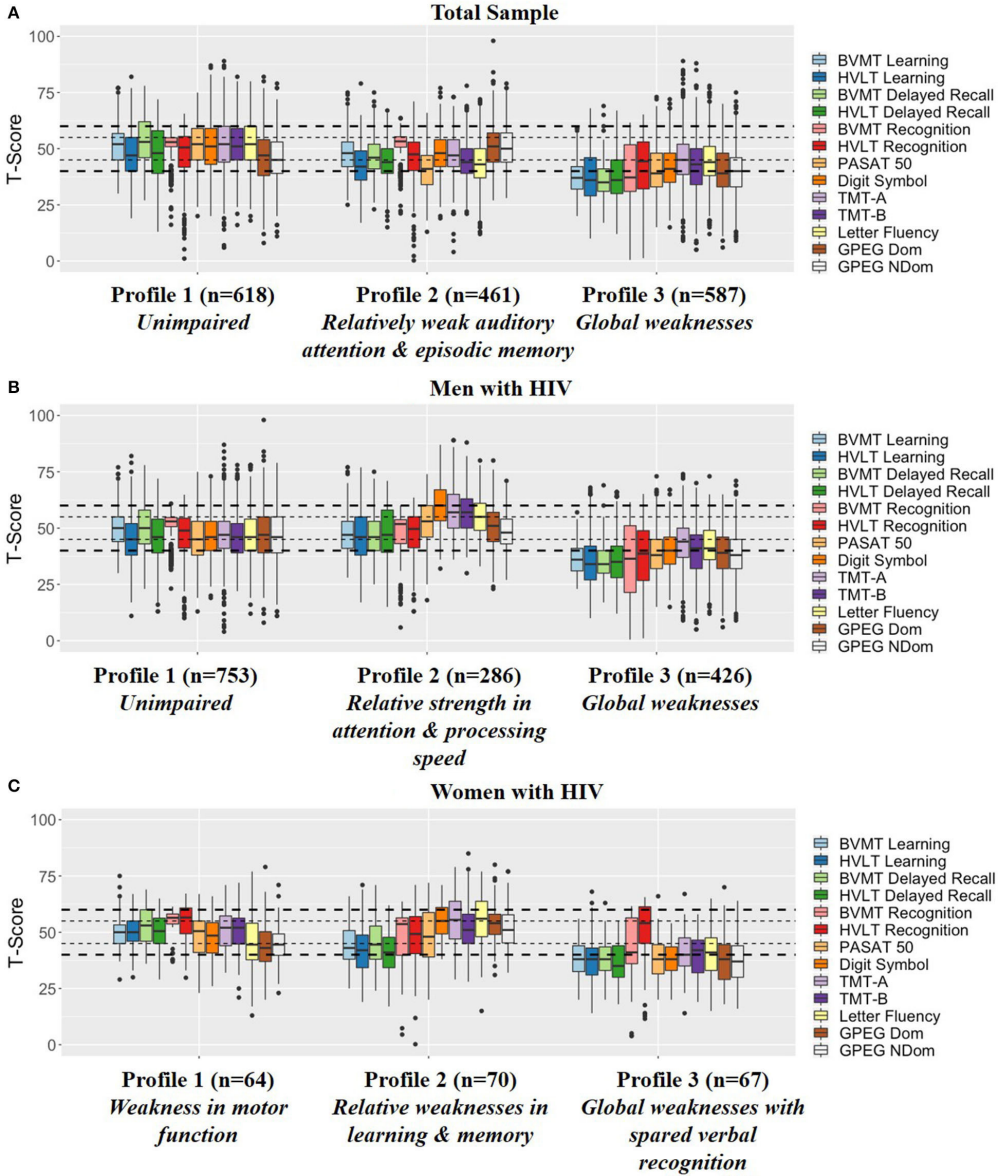
Profiling results in the **(A)** Total sample, **(B)** Men with HIV, and **(C)** Women with HIV. The small dotted line indicates a half of a standard deviation above and below the mean whereas the large dotted line indicates a full standard deviation above and below the mean. BVMT, Brief Visuospatial Memory Test-Revised; HVLT, Hopkins Verbal Learning Test Revised; PASAT, Paced Auditory Serial Addition Task; TMT, Trail Making Test; GPEG, Grooved pegboard.

Profiling of the 1,666 PWH resulted in three total groups using an ellipsoidal multivariate mixture model with equal orientation with an entropy of 0.982 (Figure 1A).

- **Profile 1 (*n* =** **618):** ***Unimpaired*** indicated by the average T-score for all NP outcomes falling into the normal/average range between 45 and 55.
- **Profile 2 (*n* =** **461):** ***Relatively weak auditory attention and episodic memory*** indicated by weaknesses in HVLT-R (learning and delayed recall), Letter Fluency, and PASAT.
- **Profile 3 (*n* =** **587):** ***Global weaknesses*** indicated by average T-scores in the impaired range on all BVMT-R outcomes, HVLT-R learning and delayed recall, GPEG-non-dominant, and PASAT as well as weaknesses on TMT-Part B, Letter Fluency, GPEG-dominant, and Digit Symbol.

**Figure 2:**
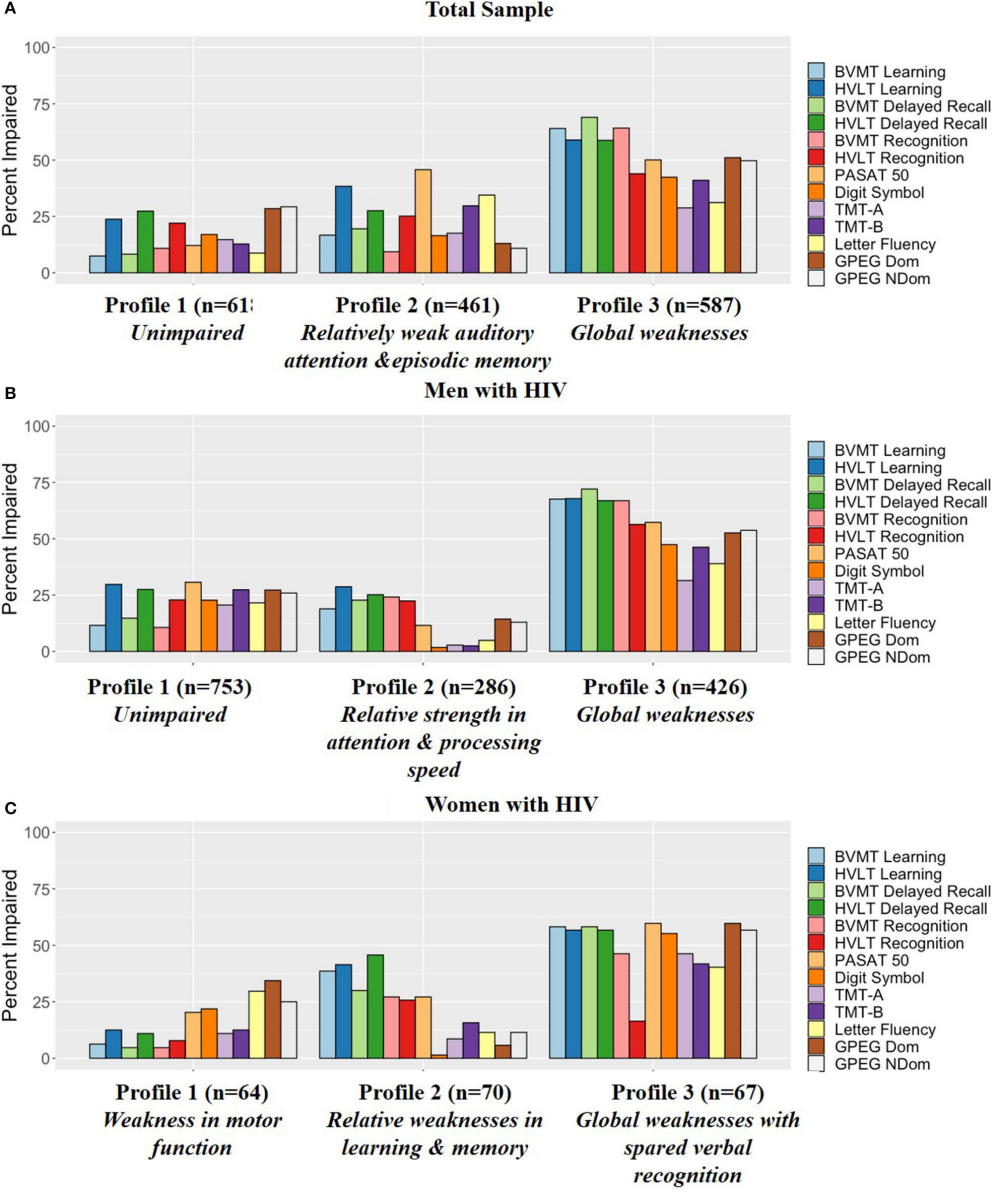
Percent impairment (T-score cutoff of 40) for each profile in the **(A)** Total sample, **(B)** Men with HIV, and **(C)** Women with HIV. BVMT, Brief Visuospatial Memory Test-Revised; HVLT, Hopkins Verbal Learning Test Revised; PASAT, Paced Auditory Serial Addition Task; TMT, Trail Making Test; GPEG, Grooved pegboard.

Figure 2A provides the percent impairment on each task within each of the profiles. Supplementary Table 1 also provides T-scores and percent impairment on each task within each of the profiles for reference.

### Identification of Cognitive Profiles in MWH and WWH Separately

Profiling of the 1,465 MWH also resulted in three groups using an ellipsoidal multivariate mixture model with equal orientation with an entropy of 0.993 (Figure 1B).

- **Profile 1 (*n* =** **753):** ***Unimpaired*** indicated by the average T-score for all NP outcomes falling into the normal range between 45 and 55.
- **Profile 2 (*n* =** **286):** ***Unimpaired with relative strength in attention and processing speed*** indicated by relative strengths (T-scores above 55) on TMT-Part A&B and Digit Symbol compared to Profile 1. Similar to Profile 1, all other NP outcomes fell into the normal range between 45 and 55.
- **Profile 3 (*n* =** **426):** ***Global weaknesses*** indicated by impairment on all BVMT-R, HVLT-R and GPEG outcomes and weaknesses on TMT-Part A&B, Letter Fluency, and Digit Symbol.

Profiling of the 201 WWH also resulted in three groups using an ellipsoidal multivariate mixture model with equal orientation with an entropy of 0.989 (Figure 1C).

- **Profile 1 (*n* =** **64):** ***Weakness in motor function*** indicated by the average T-score falling into the normal/average range (>45) or above average (>55) on all tests except for GPEG.
- **Profile 2 (*n* =** **67):** ***Relative weaknesses in learning and memory*** indicated by weaknesses on learning and delayed recall on the BVMT-R and HVLT-R with the average T-scores for the other outcomes falling in the normal range (>45).
- **Profile 3 (*n* =** **70):** ***Global weaknesses with spared verbal recognition*** indicated by average T-scores in the impaired range on learning and delayed recall on the BVMT-R and HVLT-R, GPEG, PASAT, and Digit Symbol, and weaknesses on BVMT-R recognition, TMT, and Letter Fluency. Notably, average recognition on the HVLT-R was in the normal range (M = 47.1, SD = 14.5).

Figure 2B provides the percent impairment on each task within each of the profiles within MWH and Figure 2C in WWH. Supplementary Tables 2, 3 also provide T-scores and percent impairment on each task within each of the profiles for reference.

### Predictors of Cognitive Profiles in the Total Sample

**Figure 3:**
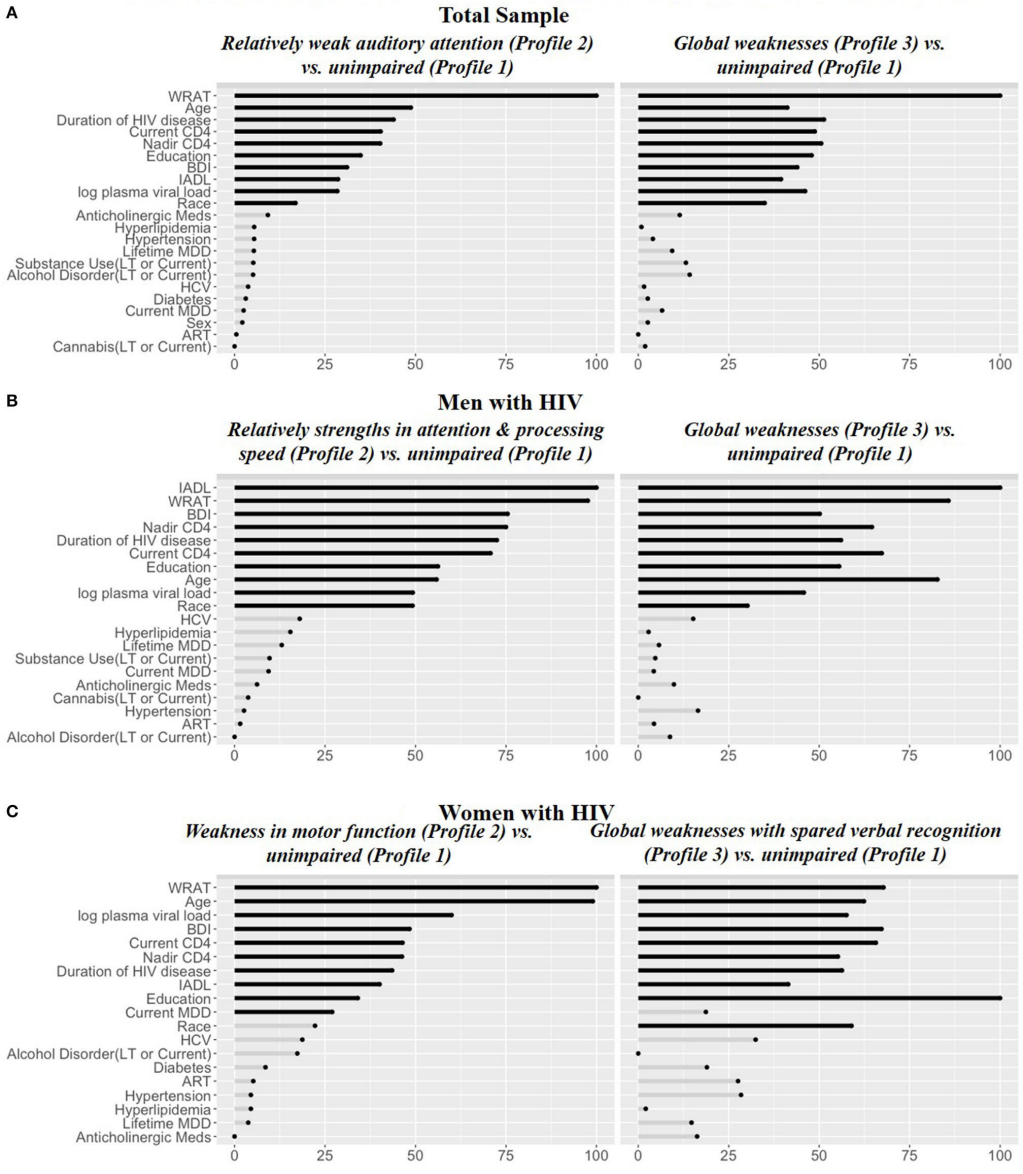
Random forest variable importance from the models for **(A)** Total sample, **(B)** Men with HIV, and **(C)** Women with HIV. IADL, Instrumental Activities of Daily Living; WRAT, Wide Range Achievement Test-4 Reading subtest; BDI, Beck Depression Inventory; LT, lifetime; HCV, Hepatitis C co-infectious; MDD, major depressive disorder.

In RF models, the top 10 variables distinguishing each of the impaired profiles—*Relatively weak auditory attention and episodic memory* [Profile 2; receiver operating curve (ROC) = 0.94] and *Global weaknesses* (Profile 3; ROC = 0.95)—from the unimpaired profile (Profile 1) were the same and included: WRAT-4, age, duration of HIV disease, nadir CD4 counts, education, BDI-II, IADL dependence, log plasma viral load, and race/ethnicity (Figure 3A). In each case, the impaired profiles or those with weaknesses (2 and 3) had lower WRAT-4 and higher BDI-II scores than the unimpaired profile (Profile 1) (Table 3). The *Relatively weak auditory attention and episodic memory* profile (Profile 2) group also was less educated, more likely to be Hispanic, and had a shorter duration of HIV disease compared to the unimpaired profile (Profile 1). However, the *Global Weaknesses* profile (Profile 3) was older, had more IADL dependence, a longer duration of HIV disease, and lower current and nadir CD4 counts as compared to the unimpaired profile.

**Table 3:** Demographic, behavioral, and clinical characteristics in the total sample of people with HIV by cognitive profile.

	**Profile 1**	**Profile 2**	**Profile 3**	
	**Unimpaired** **(*n* = 618)** ***n* (%)**	**Relatively weak auditory attention and episodic memory** **(*n* = 461)** ***n* (%)**	**Global weaknesses** **(*n* = 587)** ***n* (%)**	***P*-value**
Age, M (SD)	41.2 (9.1)	39.4 (9.4)	44.2(10.2)	<0.001
Male	544 (88)	401 (87)	520 (89)	0.73
Years of education, M (SD)	13.5 (2.5)	12.9 (2.7)	13.5 (2.8)	<0.001
WRAT-4, M (SD)	102.2 (11.9)	96.5 (13.6)	97.6 (14.2)	<0.001
Race				<0.001
White	387 (63)	202 (44)	382 (65)	
Black	119 (19)	104 (23)	91 (15)	
Hispanic	76 (12)	132 (29)	91 (15)	
Other	36 (6)	23 (5)	23 (4)	
IADL dependence	2.2 (2.7)	2.2 (2.7)	3.0 (3.1)	<0.001
BDI-II	12.4 (9.9)	13.4 (10.8)	14.7(10.4)	<0.001
**DSM-IV (CIDI) diagnoses**				
MDD				
Current	67 (17)	59 (16)	98 (22)	0.06
Lifetime	198 (51)	154 (43)	220 (50)	0.05
Alcohol (current or lifetime^*^)	19 (5)	25 (7)	28 (6)	0.47
Cannabis (current or lifetime^*^)	23 (6)	21 (6)	19 (4)	0.52
Substance use (current or lifetime^*^)	232 (76)	208 (72)	251 (71)	0.41
Anticholinergic medication	127 (21)	125 (27)	170 (29)	0.002
Hypertension	123 (21)	71 (16)	140 (25)	0.002
Hyperlipidemia	82 (14)	44 (10)	93 (16)	0.008
Diabetes	38 (6)	14 (3)	30 (5)	0.05
HCV	111 (19)	81 (18)	125 (22)	0.18
Log plasma viral load, M (SD)	3.2 (1)	3.0 (1.3)	3.1 (1.4)	0.05
CD4 count, M (SD)				
Current	434.6 (283.1)	449.9 (305.6)	406 (303.5)	0.05
Nadir	245.1 (214.7)	259.8 (251.2)	197.5 (198.3)	<0.001
Duration of HIV disease, M (SD)	8.9 (7.8)	7.9 (7.2)	10.2 (8.2)	<0.001
AIDS diagnosis	331 (54)	253 (55)	385 (66)	<0.001
On ART	322 (54)	275 (61)	369 (65)	<0.001

ART, antiretroviral therapy; WRAT-4, Wide Range Achievement Test-4th edition; IADL, Instrumental Activities of Daily Living; BDI, Beck depression inventory; CIDI, Composite International Diagnostic Interview; HCV, Hepatitis C;

* *Current or Lifetime Use disorder (abuse or dependence)*.

### Predictors of Cognitive Profiles in MWH and WWH Separately

#### MWH

In RF models in MWH, the top 10 variables distinguishing the *Global weaknesses* profile (Profile 3) and *Relative strength in attention and processing speed* (Profile 2) from the unimpaired profile (Profile 1) (ROC = 0.95 and ROC = 0.92, respectively) were the same and included: IADL dependence, WRAT-4, BDI-II, nadir CD4 count, duration of HIV disease, current CD4 count, education, age, log plasma viral load, and race (Figure 3B). The *Global weaknesses* profile (Profile 3) was older, had higher IADL dependence, and BDI-II scores, longer duration of HIV disease, and lower current and nadir CD4 counts compared to the unimpaired profile (Profile 1) (Table 4). The *Relative strength in attention and processing speed* (Profile 2) was more likely to be White, had higher WRAT-4 and lower BDI-II scores, had higher current and nadir CD4 counts, and lower log plasma viral loads than the unimpaired profile (Profile 1).

**Table 4:** Demographic, behavioral, and clinical characteristics in the sample of men with HIV by cognitive profile.

	**Profile 1**	**Profile 2**	**Profile 3**	
	**Unimpaired** **(*n* = 753)** ***n* (%)**	**Relative strength in attention and processing speed** **(*n* = 286)** ***n* (%)**	**Global weaknesses** **(*n* = 426)** ***n* (%)**	***P*-value**
Age, M (SD)	40.6 (9.1)	41.9 (9.8)	44.3(10.6)	<0.001
Years of education, M (SD)	13.4 (2.52)	13.7 (2.6)	13.6 (2.9)	0.245
WRAT-4, M (SD)	99.7 (12.8)	104.1 (11.8)	96.8 (14.5)	<0.001
Race				0.01
White	432 (57)	188 (66)	270 (63)	
Black	146 (19)	48 (17)	65 (15)	
Hispanic	139 (19)	31 (11)	76 (18)	
Other	36 (5)	19 (6)	15 (4)	
IADL complaints	2.2 (2.7)	1.4 (1.9)	3.4 (3.3)	<0.001
BDI	13.1 (10.4)	10.5 (9.)	16.2 (10.7)	<0.001
**CIDI diagnoses**				
MDD				
Current	105 (19)	21 (10)	77 (24)	<0.001
Lifetime	259 (47)	92 (45)	159 (49)	0.66
Alcohol (current or lifetime^*^)	40 (7)	3 (1)	23 (7)	0.009
Cannabis (current or lifetime^*^)	36 (7)	8 (4)	16 (5)	0.31
Substance use (current or lifetime^*^)	321 (74)	121 (73)	183 (72)	0.90
Anticholinergic medication	195 (26)	48 (17)	124 (29)	<0.001
Hypertension	140 (19)	51 (19)	107 (26.)	0.01
Hyperlipidemia	96 (13)	40 (15)	57 (14)	0.82
Diabetes	40 (5)	8 (3)	22 (5)	0.22
HCV	125 (17)	46 (17)	88 (21)	0.14
Log plasma viral load, M (SD)	3.2 (1.3)	2.9 (1.3)	3.2 (1.5)	0.01
**CD4 count, M (SD)**				
Current	432.4 (301.1)	479.0 (272.8)	381.4 (295.0)	<0.001
Nadir	238.6 (232.8)	268.2 (209.8)	188.8 (196.1)	<0.001
Duration of HIV disease, M (SD)	8.9 (7.8)	8.5 (7.4)	10.4 (8.6)	0.01
AIDS diagnosis	440 (56)	113 (47)	281 (66)	<0.001
On ART	426 (58)	166 (59)	265 (64)	0.10

ART, antiretroviral therapy; WRAT-4, Wide Range Achievement Test; IADL, Instrumental Activities of Daily Living; BDI, Beck depression inventory; CIDI, Composite International Diagnostic Interview; HCV, Hepatitis C;

* *Current or Lifetime Use disorder (abuse or dependence)*.

#### WWH

In RF models in WWH, nine out of the top 10 variables distinguishing the *Relative weaknesses in learning and memory* profile (Profile 2) and *Global weakness with spared verbal recognition* (Profile 3) from the profile only demonstrating *Weakness in motor function* (Profile 1; ROC = 0.95 and ROC = 0.90, respectively) were the same and included: WRAT-4, age, log plasma viral load, BDI-II, current and nadir CD4 count, duration of HIV infection, IADL dependence, and education (Figure 3C). The only unique variable distinguishing the profile demonstrating *Relative weaknesses in learning and memory* (Profile 2) from the profile demonstrating *Weakness in motor function* (Profile 1) was race. However, the unique variable distinguishing the profile demonstrating *Global weaknesses with spared verbal recognition* (Profile 3) from the profile demonstrating *Weakness in motor function* (Profile 1) was a current diagnosis of MDD. The *Relative weaknesses in learning and memory* profile (Profile 2) was older, less likely to be Hispanic, more likely to be non-Hispanic White, and had lower BDI-II scores compared the profile demonstrating *Weakness in motor function* (Profile 1) (Table 5). However, the *Global weaknesses with spared verbal recognition* profile (Profile 3) was more likely to be non-Hispanic White, have lower WRAT-4 scores, had more IADL dependence and higher BDI-II scores, was more likely to have a current diagnosis of MDD, have higher log plasma viral loads, lower current and nadir CD4 counts, and a shorter duration of HIV disease compared to the profile demonstrating *Weakness in motor function* (Profile 1).

**Table 5:** Demographic, behavioral, and clinical characteristics in the sample of women with HIV by cognitive profile.

	**Profile 1**	**Profile 2**	**Profile 3**	
	**Weakness in motor function**	**Relative weaknesses in learning and memory**	**Global weaknesses with spared verbal recognition**	***P*-value**
	**(*n* = 64)**	**(*n* = 70)**	**(*n* = 67)**	
	***n* (%)**	***n* (%)**	***n* (%)**	
Age, M (SD)	37.4 (8.5)	42.4 (9.9)	41.9 (9.8)	0.005
Years of education, M (SD)	11.5 (2.5)	12.6 (2.4)	11.9 (2.6)	0.03
WRAT-4, M (SD)	96.6 (9.8)	95.5 (13.9)	88.9 (13.5)	0.003
Race				0.007
White	21 (33)	32 (46)	28 (42)	
Black	16 (25)	26 (37)	13 (19)	
Hispanic	25 (39)	8 (11)	20 (30)	
Other	2 (3)	4 (6)	6 (9)	
IADL dependence	2.7 (3.0)	2.2 (2.7)	3.8 (3.5)	0.01
BDI-II	13.7 (9.5)	10.9 (9.2)	16.0 (10.5)	0.01
**DSM-IV (CIDI) diagnoses**				
MDD				
Current	5 (14)	2 (5)	14 (34)	0.003
Lifetime	17 (47)	17 (45)	28 (68)	0.06
Alcohol (current or lifetime^*^)	3 (8)	2 (5)	1 (2)	0.51
Cannabis (current or lifetime^*^)	1 (3)	2 (5)	0 (0)	0.34
Substance use (current or lifetime^*^)	22 (81)	21 (66)	21 (67)	0.36
Anticholinergic medication	16 (25)	18 (26)	21 (31)	0.66
Hypertension	8 (12)	16 (23)	12 (18)	0.28
Hyperlipidemia	5 (8)	9 (13)	12 (18)	0.21
Diabetes	5 (8)	2 (3)	58	0.4
HCV	22 (34)	18 (26)	18 (27)	0.53
Log plasma viral load, M (SD)	3.1 (0.9)	3.1 (1.2)	3.2 (1.3)	0.72
CD4 count, M (SD)				
Current	448.4 (282.9)	516.5 (309.6)	358.2 (304.7)	0.01
Nadir	298.6 (269.1)	273.1 (232.0)	181.3 (173.5)	0.009
Duration of HIV disease, M (SD)	7.3 (5.4)	7.41 (6.1)	6.5 (5.3)	0.76
AIDS diagnosis	32 (50)	36 (51)	47 (70)	0.03
On ART	34 (55)	36 (52)	39 (61)	0.58

ART, antiretroviral therapy; WRAT-4, Wide Range Achievement Test; IADL, Instrumental Activities of Daily Living; BDI, Beck depression inventory; CIDI, Composite International Diagnostic Interview; HCV, Hepatitis C;

* *Current or Lifetime Use disorder (abuse or dependence)*.

## Discussion

In this large-scale study using a novel pipeline combination of machine learning methods, we provide further evidence in support of heterogeneity in cognitive function among PWH. Our results do not negate the heterogeneity in cognitive function in HIV-uninfected individuals but rather highlights the heterogeneity among PWH that can often be masked by a dichotomous HAND categorization. In the total sample, we identified an unimpaired profile, a profile of relatively weak auditory attention and episodic memory, and a global weakness profile. As expected, given the relative sample sizes, the cognitive patterns in the total sample were in greater alignment with those found among MWH compared to WWH. Similar to results in the overall sample, we identified an unimpaired profile and a global weakness profile in MWH; however, unlike the overall sample and inconsistent with hypotheses of domain-specific cognitive impairment profiles in both MWH and WWH, MWH demonstrated a profile with relative strengths in attention and processing speed. Conversely, there were no unimpaired, cognitive strength or global weakness profiles among WWH. Rather, as hypothesized WWH demonstrated cognitive profiles reflecting a global weakness (with spared verbal recognition) and domain-specific impairment including a weakness in learning and memory and motor skills. These findings suggest that sex and the sociodemographic factors associated with female sex within the HIV-infected population contribute to the heterogeneity in cognitive function among PWH. Studies examining cognitive function in combined samples of men and women may mask important sex differences in cognitive functioning among PWH, particularly in male-dominant samples such as the current sample. These sex differences in cognitive profiles among PWH may result from biological sex differences and/or the psychosocial factors that tend to characterize WWH more than MWH (e.g., low education, poverty). Biological sex differences include those seen in the general population such as sex steroid hormones (e.g., estrogen, progesterone, testosterone), female-specific reproductive events (e.g., parity, reproductive span, hormone therapies) and genetic factors or previously-reported sex differences specifically in HIV disease characteristics unmeasured herein (e.g., size of viral reservoirs, CD4 cell count at seroconversion) (47, 48). Regardless of the underlying mechanism, characterizing these sex differences in cognitive functioning among PWH can provide inroads to identifying mechanisms of cognitive dysfunction and optimizing risk assessments and diagnostic and therapeutic strategies for each sex.

A notable sex difference in profiles was the lack of the unimpaired or cognitive strength profile among WWH that was observed among MWH. Our cognitive profile analyses are in line with prior studies that suggests that WWH are often but not always, more likely to demonstrate cognitive deficits than MWH (10). Our analysis suggests that the impairment manifests more often as domain-specific impairment (i.e., learning, memory, motor) in women than in men that may not be revealed in a more cross-domain summary measure like GDS or global T-scores. This female vulnerability to cognitive deficits is thought to reflect sociodemographic differences whereby low education and socioeconomic status and their associated psychosocial risk factors (e.g., depression, poverty, early-life trauma, barriers to health care, co-infections) are more prevalent among WWH vs. MWH (10, 22, 49). These psychosocial risk factors can have adverse effects on the brain that lower cognitive reserve (23, 24, 50, 51), suggesting that interventions geared toward addressing these psychosocial factors should be a priority for WWH and/or for women who are at increased risk of HIV. In support of these studies, Sundermann et al. (17) found that the higher rates of cognitive impairment in WWH vs. MWH were eliminated after adjusting for the lower reading level (i.e., WRAT-4 score) that characterized WWH compared to MWH. Biological differences may also contribute to sex differences in the pattern and magnitude of cognitive impairment in PWH including disease characteristics, brain structure/function, sex steroid hormones and female-specific hormonal milieus (e.g., pregnancy, menstrual cycle, menopause transition). There is also evidence to suggest that WWH may be more cognitively susceptible than MWH to the effects of mental health factors (25).

As mentioned, only women demonstrated more domain-specific cognitive profiles including weakness in motor functioning and relative weakness in learning and memory. Similarly, previous studies report that learning, memory, and motor functioning are among the domains in which cognitive impairment is more common among WWH vs. MWH (10) and these differences persisted after adjusting for HIV RNA and CD4 counts (21). These sex differences in domain-specific impairment may reflect psychosocial factors (e.g., cognitive reserve, mental health), biological factors (sex steroid hormones, genetic), or interactions among them. Although women in general demonstrate relative advantages in verbal memory and fine motor function compared men (52–57) likely due, at-least in part, to the effects of estrogen on the developing brain and the neuroprotective effects of circulating estradiol (58–60), the menopause transition has been associated with declines in verbal memory and motor function (61–63). The mean age of women in our study was 41 (SD = 9.6; 33% >45 years of age) suggesting that a portion of women may be experiencing cognitive deficits associated with reproductive aging. Germane to the learning/memory impairment in WWH, women are more vulnerable to the negative effects of stress hormones on hippocampal-dependent tests compared to men (64). This finding may be particularly relevant to the current sample considering the high prevalence of psychosocial stressors among WWH including childhood trauma and domestic violence (65).

Unlike MWH, WWH demonstrated a global impairment profile with spared verbal recognition. Consistently, previous findings regarding memory impairment among PWH found this impairment to be more dependent on frontal and subcortical structures with relatively normal memory retention but impaired memory retrieval (recall but not recognition deficits) (66–68). Even in the female-specific profile of relative weakness in learning and memory, recognition was less impaired compared to learning and recall. We can only speculate as to why the sparing of recognition in the global impairment profile was specific to WWH and to verbal vs. visual memory. It is possible that, in the context of cognitive impairment in HIV, the female advantage in verbal memory may be most salient for the least cognitively-taxing memory component, recognition performance, and this advantage is not fully adjusted for in our demographically-corrected T-scores.

Despite the heterogeneity in cognitive profiles by sex, the sociodemographic/clinical/biological factors associated with these cognitive profiles were similar for MWH and WWH suggesting that, although the same factors confer increased vulnerability to cognitive dysfunction, the adverse effects of these factors impact brain function differently in men and women. In both MWH and WWH, WRAT-4 had the greatest discriminative value of profile class followed by HIV disease variables (e.g., CD4 count, viral load and estimated duration of HIV disease), depressive symptoms, age, race/ethnicity and years of education. WRAT-4 scores have been consistently identified as an important determinant of cognitive function among PWH, with lower WRAT-4 scores conferring risk for cognitive impairment (17, 69). WRAT-4 performance may be particularly salient in this population, given that reading level may reflect education quality, above and beyond years of education, especially in lower socioeconomic populations because of the many factors impacting education quality (e.g., ability to attend school, economic disadvantages in schools within low SES districts) (69). Additionally, reading level is associated with health outcomes including hospitalizations and outpatient doctor visits (70) and, thus, may be a proxy for biopsychosocial factors underlying general health (e.g., socioeconomic status, self-efficacy).

HIV disease variables were also strong determinants of cognitive profiles in both men and women. Aside from some instances of a shorter duration of HIV disease relating to more cognitive impairment in WWH and in the total sample, the more biologically-based HIV disease variables were associated with cognitive impairment in the expected direction; higher current and nadir CD4 count and lower viral load were protective against cognitive impairment. It is curious that the global weakness with spared verbal recognition profile in women was associated with more severe HIV-related variables (i.e., higher viral loads, lower current, and nadir CD4 counts) yet with shorter duration of HIV infection. We speculate that the shorter HIV infection in WWH may reflect CNS effects of untreated and/or early-course HIV infection. Alternatively, the self-reported shorter duration of infection may not have been accurate, to the extent that WWH lived longer with untested/undetected infections. Findings are consistent with a wealth of literature relating proxies of HIV disease burden and severity to cognitive function (71–73) and suggests that, even in the era of effective ART when viral suppression is common, HIV disease burden can have adverse effects on the brain possibly due to poor penetration of ARTs into the CNS, ART resistance, poor medication adherence (74), and/or the establishment of viral reservoirs in the CNS reservoir (75, 76).

In line with hypotheses of mental health factors relating to cognitive impairment profiles more strongly in women, current diagnosis of MDD was a predictor of cognitive profiles only among WWH. Although the prevalence of a current or lifetime diagnosis of MDD did not differ between WWH and MWH, MDD was an important risk factor of demonstrating *Global weaknesses with spared verbal recognition* (Profile 2) compared to the profile demonstrating only *Weakness in motor function* (Profile 1). This finding aligns with our work demonstrating that MDD may have a greater impact in women compared to men (25). Our work indicates that HIV comorbid with depression affects certain cognitive domains including cognitive control, and that these effects are largest in women. Specifically, WWH with elevated depressive symptoms had 5 times the odds of impairment on Stroop Trial 3, a measure of behavioral inhibition, compared to HIV-uninfected depressed women, and 3 times the odds of impairment on that test compared to depressed MWH. In a recent meta-analysis, small to moderate deficits in declarative memory and cognitive control were documented not only in individuals with current MDD but also in individuals with remitted MDD, leading to the conclusion that these deficits occur independently of episodes of low mood in individuals with “active” MDD (77). Together these lines of work suggest that MDD would exacerbate (or co-occur with factors that cause) cognitive difficulties in PWH, particularly in the cognitive domains of declarative memory and cognitive control in WWH.

Our study has limitations. Although we were adequately powered within both WWH and MWH (10), the magnitude of power was discrepant by sex considering that women represented 20% of our sample. Larger-scale studies in WWH only are currently underway. The generalizability of our findings also warrant additional study as the profiles identified here may not represent the profiles among all PWH. Due to the unavailability of data, we were unable to explore certain psychosocial factors (e.g., early life trauma, perceived stress) as potential determinants of cognitive profiles. Our analyses were cross-sectional which allows us to identify determinants associated with cognitive profiles but precludes us from determining the temporal relationships between these factors and cognitive function. Although many of the related factors may be risk factors for cognitive impairment, reverse causality is possible with some of the factors resulting from cognitive impairment (e.g., depression, IADL). Additionally, interpretation of the machine learning results should be done with care as RF is an ensemble model that is inherently non-linear in nature. This means that the importance and predictive power of every variable is specified in the context of other variables. This can lead to situations where an important predictive variable in the RF model has no significant difference in the overall comparison but has dramatic differences when included with other variables in the model. As such, this model should be interpreted as hypothesis-generating and identifies variables in need of further investigation. Lastly, because our study was focused on sex differences in cognitive profiles within PWH, we did not include a HIV-seronegative comparison group. Thus, we cannot determine the degree to which HIV contributes to sex differences in cognitive profiles. However, the independent HIV-related predictors does suggest that HIV has a role. Despite these limitations, we selected RF over linear models such as lasso and ridge regression because RF models had more predictive power and higher accuracy in this data compared to the linear models, even linear models with tuning parameters such as ridge and lasso that can used for feature selection. The results from these models mirror the *P*-values for the univariate comparisons (see Tables 1–5), which is expected since analysis of variance and *t*-tests are also linear models. Moreover, RF models are more optimal for handling missing data, the inclusion of categorical predictor variables, and the use of categorical outcome measures which was the case in the present study. RF models also account for the complexity in the data that can arise from multicollinearity often seen in large feature sets.

In conclusion, our results also suggest that sex is a contributor to the heterogeneity in cognitive profiles among PWH and that cognitive findings from MWH or male-dominant samples cannot be wholly generalized to WWH. Whereas, MWH showed an unimpaired profile and even a cognitively advantageous profile, WWH only showed impairment profiles that included global and more domain-specific impairment, which supports previous findings of greater cognitive impairment in WWH than in MWH (10). Although the strongest determinants of cognitive profiles were similar in MWH and WWH including WRAT-4, HIV disease characteristics, age and depressive symptoms, the direction of these associations sometimes differed. This suggests that the effects of certain biological, clinical, or demographic factors on the brain and cognition may manifest differently in MWH and WWH and that sex may contribute to heterogeneity not only in cognitive profiles but in their determinants although studies with larger numbers of WWH are needed to more definitively test these hypotheses. It is important to detect these differing cognitive profiles and their associated risk/protective factors as this information can help to identify differing mechanisms contributing to cognitive impairment and whether these mechanisms are related to HIV disease, neurotoxic effects of ART medications, and/or comorbidities that are highly prevalent among PWH (e.g., depression, substance abuse, hyperlipidemia). Given the longer lifespan of PWH in the era of effective antiretroviral therapy, cognitive profiling will also inform aging-related effects on cognition in the context of HIV and perhaps early clinical indicators of age-related neurodegenerative disease. By identifying cognitive profiles and their underlying mechanisms, we can ultimately improve our ability to treat by tailoring and directing intervention strategies to those most likely to benefit. Overall, our results stress the importance of considering sex differences in studies of the pathogenesis, clinical presentation, and treatment of cognitive dysfunction in HIV.

## Data Availability Statement

The data analyzed in this study is subject to the following licenses/restrictions: data from our study are available upon request. Persons with HIV are highly stigmatized, and even in the presence of strong de-identification, the risk of re-identification is real. Protecting persons with HIV remains our top priority. The Data Access Committee whom imposed these restrictions and to whom data requests should be made is the HNRP Data Resource Committee. Requests to access these datasets should be directed to hnrpresource@ucsd.edu.

## Ethics Statement

The studies involving human participants were reviewed and approved by the Institutional Review Board of the University of California, San Diego. The patients/participants provided their written informed consent to participate in this study.

## Author Contributions

ES aggregated the data. LR, RD, and EP conducted statistical analyses. LR and ES have primary responsibility for final content and wrote the paper. All authors contributed to the project conception, design, manuscript editing, statistical review, and read and approved the final manuscript.

## Conflict of Interest

The authors declare that the research was conducted in the absence of any commercial or financial relationships that could be construed as a potential conflict of interest. The handling editor is currently organizing a Research Topic with one of the authors ES.
