# Supplementary material for: Sex Differences in the Patterns and Predictors of Cognitive Function in HIV
Source: Front Neurol. 2020 Nov 23;11:551921. doi: 10.3389/fneur.2020.551921 (PMC7732436; doi:10.3389/fneur.2020.551921)
Supplement: Supplementary file 1 [file Table_1.DOCX]

**Supplemental Table 1.** Neuropsychological test performance by cognitive profile in the total sample of people with HIV.

|  | Profile 1 | Profile 2 | Profile 3 |  |
| --- | --- | --- | --- | --- |
|  | Unimpaired | Relatively Weak Auditory Attention & Episodic memory | Global weaknesses |  |
|  | (n=618) | (n=461) | (n=587) |  |
|  | n (%) | n (%) | n (%) | *P*-value |
| ***T-scores*** | M (SD) | M (SD) | M (SD) |  |
| BVMT-R |  |  |  |  |
| Total Learning | 51.49 (8.69) | 47.36 (8.14) | 37.48 (7.07) | <0.001 |
| Delayed Recall | 52.99 (9.55) | 47.07 (9.05) | 35.69 (7.31) | <0.001 |
| Recognition | 51.34 (6.46) | 51.84 (6.26) | 34.16 (20.09) | <0.001 |
| HVLT-R |  |  |  |  |
| Total Learning | 47.53 (11.11) | 42.60 (8.96) | 37.42 (11.76) | <0.001 |
| Delayed Recall | 47.61 (11.67) | 44.54 (8.63) | 37.52 (11.46) | <0.001 |
| Recognition | 47.66 (10.76) | 45.45 (11.62) | 38.99 (18.69) | <0.001 |
| Grooved Pegboard |  |  |  |  |
| Dominant | 46.24 (11.86) | 51.17 (9.01) | 40.11 (11.12) | <0.001 |
| Non-dominant | 45.51 (10.97) | 50.43 (9.37) | 39.35 (10.75) | <0.001 |
| Trail Making Test |  |  |  |  |
| Part A | 51.81 (12.52) | 47.38 (9.67) | 45.37 (11.92) | <0.001 |
| Part B | 51.88 (10.72) | 44.26 (9.06) | 42.06 (12.17) | <0.001 |
| Letter Fluency | 52.26 (10.09) | 43.07 (11.19) | 44.55 (10.98) | <0.001 |
| PASAT 50 | 51.63 (10.07) | 40.84 (9.11) | 40.48 (11.37) | <0.001 |
| Digit Symbol Test | 51.32 (11.82) | 48.41 (9.20) | 42.07 (10.06) | <0.001 |
| Global deficit score | 0.31 (0.37) | 0.41 (0.32) | 0.92 (0.74) | <0.001 |
| ***Percent impairment* (40 cutpoint)** | N (%) | n (%) | n (%) |  |
| BVMT-R |  |  |  |  |
| Total Learning | 46 (7) | 77 (17) | 376 (64) | <0.001 |
| Delayed Recall | 51 (8) | 90 (19) | 405 (69) | <0.001 |
| Recognition | 67 (11) | 43 (9) | 377 (64) | <0.001 |
| HVLT-R |  |  |  |  |
| Total Learning | 147 (24) | 177 (38) | 346 (59) | <0.001 |
| Delayed Recall | 169 (27) | 240 (52) | 345 (59) | <0.001 |
| Recognition | 136 (22) | 186 (40) | 258 (44) | <0.001 |
| Grooved Pegboard |  |  |  |  |
| Dominant | 176 (28) | 123 (27) | 300 (51) | <0.001 |
| Non-dominant | 181 (29) | 140 (30) | 292 (50) | <0.001 |
| Trail Making Test |  |  |  |  |
| Part A | 91 (15) | 168 (36) | 169 (29) | <0.001 |
| Part B | 79 (13) | 137 (30) | 241 (41) | <0.001 |
| Letter Fluency | 54 (9) | 159 (34) | 183 (31) | <0.001 |
| PASAT 50 | 75 (12) | 211 (46) | 294 (50) | <0.001 |
| Digit Symbol Test | 105 (17) | 76 (16) | 249 (42) | <0.001 |

M=mean; SD=standard deviation; BVMT-R=Benton Visual Retention Test-Revised; HVLT-R=Hopkins Verbal Learning Test-Revised; PASAT=Paced Auditory Serial Addition Test

**Supplemental Table 2.** Neuropsychological test performance by cognitive profile in the sample of men with HIV.

|  | Profile 1 | Profile 2 | Profile 3 |  |
| --- | --- | --- | --- | --- |
|  | Unimpaired | Relative strength in attention & processing speed | Global weaknesses |  |
|  | (n=753) | (n=286) | (n=426) |  |
|  | n (%) | n (%) | n (%) | *P*-value |
| ***T-scores*** | M (SD) | M (SD) | M (SD) |  |
| BVMT-R |  |  |  |  |
| Total Learning | 49.92 (8.53) | 47.79 (9.36) | 36.57 (6.66) | <0.001 |
| Delayed Recall | 50.28 (10.16) | 46.96 (9.75) | 35.05 (7.48) | <0.001 |
| Recognition | 51.36 (5.99) | 48.16 (9.34) | 31.84 (21.35) | <0.001 |
| HVLT-R |  |  |  |  |
| Total Learning | 44.98 (10.09) | 46.56 (11.89) | 35.54 (11.19) | <0.001 |
| Delayed Recall | 46.08 (10.26) | 47.30 (11.81) | 35.67 (10.82) | <0.001 |
| Recognition | 46.86 (9.70) | 47.09 (10.01) | 33.87 (19.68) | <0.001 |
| Grooved Pegboard |  |  |  |  |
| Dominant | 47.21 (11.80) | 50.29 (9.77) | 39.09 (10.55) | <0.001 |
| Non-dominant | 47.03 (11.37) | 48.09 (8.09) | 38.44 (43.54) | <0.001 |
| Trail Making Test |  |  |  |  |
| Part A | 47.09 (10.95) | 58.07 (10.76) | 43.54 (10.52) | <0.001 |
| Part B | 45.95 (9.92) | 56.50 (9.49) | 39.58 (11.09) | <0.001 |
| Letter Fluency | 47.07 (10.66) | 55.06 (9.21) | 41.48 (10.05) | <0.001 |
| PASAT 50 | 45.33 (10.86) | 52.24 (9.95) | 38.48 (10.36) | <0.001 |
| Digit Symbol Test | 46.52 (8.85) | 59.95 (9.87) | 40.20 (8.90) | <0.001 |
| Global deficit score | 0.41 (0.38) | 0.21 (0.21) | 1.05 (0.76) | <0.001 |
| ***Percent impairment* (40 cutpoint)** | N (%) | n (%) | n (%) |  |
| BVMT-R |  |  |  |  |
| Total Learning | 87 (12) | 54 (19) | 288 (67) | <0.001 |
| Delayed Recall | 239 (32) | 65 (23) | 307 (72) | <0.001 |
| Recognition | 80 (11) | 69 (24) | 285 (67) | <0.001 |
| HVLT-R |  |  |  |  |
| Total Learning | 224 (30) | 82 (29) | 289 (68) | <0.001 |
| Delayed Recall | 207 (27) | 72 (25) | 285 (67) | <0.001 |
| Recognition | 172 (23) | 64 (22) | 240 (56) | <0.001 |
| Grooved Pegboard |  |  |  |  |
| Dominant | 205 (27) | 41 (14) | 224 (53) | <0.001 |
| Non-dominant | 195 (26) | 37 (13) | 229 (54) | <0.001 |
| Trail Making Test |  |  |  |  |
| Part A | 155 (21) | 8 (3) | 134 (31) | <0.001 |
| Part B | 206 (27) | 7 (2) | 197 (46) | <0.001 |
| Letter Fluency | 162 (21) | 14 (5) | 166 (39) | <0.001 |
| PASAT 50 | 211 (31) | 33 (11) | 244 (57) | <0.001 |
| Digit Symbol Test | 171 (23) | 5 (2) | 202 (47) | <0.001 |

M=mean; SD=standard deviation; BVMT-R=Benton Visual Retention Test-Revised; HVLT-R=Hopkins Verbal Learning Test-Revised; PASAT=Paced Auditory Serial Addition Test

**Supplemental Table 3.** Neuropsychological test performance by cognitive profile in the sample of women with HIV.

|  | Profile 1 | Profile 2 | Profile 3 |  |
| --- | --- | --- | --- | --- |
|  | Weakness in motor function | Relative weaknesses in learning & memory | Global weaknesses with spared verbal recognition |  |
|  | (n=64) | (n=70) | (n=67) |  |
|  | n (%) | n (%) | n (%) | *P*-value |
| ***T-scores*** | M (SD) | M (SD) | M (SD) |  |
| BVMT-R |  |  |  |  |
| Total Learning | 50.36 (7.97) | 43.57 (9.29) | 37.88 (7.34) | <0.001 |
| Delayed Recall | 52.94 (8.40) | 46.07 (11.55) | 38.27 (7.89) | <0.001 |
| Recognition | 54.86 (5.61) | 46.47 ( 16.48) | 43.21 (15.32) | <0.001 |
| HVLT-R |  |  |  |  |
| Total Learning | 49.75 (8.11) | 42.11 (11.04) | 43.21 (15.32) | <0.001 |
| Delayed Recall | 50.29 (8.20) | 41.11 (9.51) | 36.72 (9.91) | <0.001 |
| Recognition | 54.59 (8.14) | 47.12 (14.52) | 49.82 (15.64) | <0.001 |
| Grooved Pegboard |  |  |  |  |
| Dominant | 44.27 (10.87) | 54.04 (9.44) | 37.52 (11.09) | <0.001 |
| Non-dominant | 44.66 (9.43) | 51.60 (9.90) | 36.67 (10.21) | <0.001 |
| Trail Making Test |  |  |  |  |
| Part A | 50.84 (9.49) | 55.46 (11.02) | 40.82 (9.46) | <0.001 |
| Part B | 50.23 (9.94) | 52.07 (11.80) | 39.98 (8.04) | <0.001 |
| Letter Fluency | 44.98 (12.74) | 55.00 (11.97) | 40.46 (10.45) | <0.001 |
| PASAT 50 | 48.36 (10.06) | 48.20 (12.37) | 38.22 (9.77) | <0.001 |
| Digit Symbol Test | 47.59 (9.17) | 54.69 (7.79) | 38.22 (7.87) | <0.001 |
| Global deficit score | 0.27 (0.28) | 0.29 (0.34) | 0.96 (0.62) | <0.001 |
| ***Percent impairment* (40 cutpoint)** | N (%) | n (%) | n (%) |  |
| BVMT-R |  |  |  |  |
| Total Learning | 4 (6) | 27 (39) | 39 (58) | <0.001 |
| Delayed Recall | 3 (5) | 21 (30) | 39 (58) | <0.001 |
| Recognition | 3 (5) | 19 (27) | 45 (64) | <0.001 |
| HVLT-R |  |  |  |  |
| Total Learning | 8 (12) | 29 (41) | 38 (57) | <0.001 |
| Delayed Recall | 7 (11) | 32 (46) | 38 (57) | <0.001 |
| Recognition | 5 (8) | 18 (26) | 11 (16) | <0.001 |
| Grooved Pegboard |  |  |  |  |
| Dominant | 22 (34) | 4 (6) | 40 (60) | <0.001 |
| Non-dominant | 16 (25) | 8 (11) | 38 (57) | <0.001 |
| Trail Making Test |  |  |  |  |
| Part A | 7 (11) | 6 (9) | 31 (46) | <0.001 |
| Part B | 8 (13) | 11 (16) | 28 (42) | <0.001 |
| Letter Fluency | 19 (30) | 8 (11) | 27 (40) | <0.001 |
| PASAT 50 | 13 (20) | 19 (27) | 40 (60) | <0.001 |
| Digit Symbol Test | 14 (22) | 1 (1) | 37 (55) | <0.001 |

M=mean; SD=standard deviation; BVMT-R=Benton Visual Retention Test-Revised; HVLT-R=Hopkins Verbal Learning Test-Revised; PASAT=Paced Auditory Serial Addition Test
