# Supplementary material for: Sex Differences in the Patterns and Predictors of Cognitive Function in HIV
Source: Front Neurol. 2020 Nov 23;11:551921. doi: 10.3389/fneur.2020.551921 (PMC7732436; doi:10.3389/fneur.2020.551921)
Supplement: Supplementary file 2 [file Table_2.DOCX]

---

title: "Sex Differences Profiling: SOM followed by MClust"

output:

html_document:

keep_md: yes

number_sections: yes

toc: yes

toc_float: yes

editor_options:

chunk_output_type: console

---

```{r Load_Packages, warning=FALSE, include=FALSE}

library(readxl)

library(ggplot2)

library(reshape2)

library(kohonen)

library(ggplot2)

library(factoextra)

library(xlsx)

library(mclust)

library(dplyr)

library(arsenal)

library(htmlTable)

library(tidyr)

library(ggthemes)

library(tidyverse)

library(broom)

library(dlookr)

library(kableExtra)

library(summarytools)

library(qwraps2)

library(finalfit)

library(plotly)

library(tableone)

library(compareGroups)

library(htmltools)

library(data.table)

library(gridExtra)

library(caret)

library(caretEnsemble)

library(DMwR)

library(mice)

library(VIM)

library(pROC)

##### functions to be used later

createAndPlotClustersSOM <- function(DataToCluster, clustVars, DataInfo){

s=1234

CompleteScaledData <- as.matrix(scale(DataToCluster[,clustVars]))

#Create SOM Grid

som_grid <- somgrid(xdim = 10, ydim=10, topo="hexagonal", neighbourhood.fct = "gaussian")

#som_grid <- somgrid(xdim = 10, ydim=10, topo="hexagonal") # for smaller sample sizes ()

#Train SOM

set.seed(s)

som_model <- som(CompleteScaledData,

grid=som_grid,

rlen=200,

#alpha=c(0.05,0.01),

keep.data = TRUE)

SOMCodedata <- as.data.frame(som_model$codes )

set.seed(s)

MClustered <- Mclust(SOMCodedata, G=3:20)

m.best2 <- dim(MClustered$z)[2]

cat("model-based optimal number of clusters:", m.best2, "\n")

fvizplot<- fviz_nbclust(SOMCodedata, kmeans, method = "wss", k.max = 20)

#plot(fvizplot)

#Now do the actual clustering

som_cluster<-MClustered$classification

patient_SOM = som_model$unit.classif

patient_clust = som_cluster[patient_SOM]

ClusterData<-as.data.frame(DataToCluster[,cogVars])

ClusterData$Cluster <- as.factor(patient_clust)

ClusterData$ID<-seq.int(nrow(ClusterData))

DataToCluster$Cluster<- ClusterData$Cluster

#count summary of what is in each and add to cluster names

summary(ClusterData$Cluster)

s<-summary(ClusterData$Cluster)

clusternames<-paste(1:length(s), '(n=', s,')' )

e<-mean(1-MClustered$uncertainty)

ti<- paste(DataInfo, 'n =', length(patient_clust), 'model = ', MClustered$modelName, ',Entropy =', round(e, digits = 3))

mdata <-melt(ClusterData, id = c('ID', 'Cluster'))

p <- ggplot(mdata, aes(Cluster, value, fill = variable)) + geom_boxplot() +

ylab("Value")+scale_fill_discrete(name = 'Test')+

geom_hline(yintercept=45, linetype = "dashed", size = .5)+geom_hline(yintercept=55, linetype = "dashed", size = .5)+

geom_hline(yintercept=40, linetype = "dashed", size = 1)+geom_hline(yintercept=60, linetype = "dashed", size = 1)+

scale_x_discrete(labels = clusternames) + ggtitle(ti)

ClustResults = list("SomCodeddata" = SOMCodedata, "MClustered" = MClustered, "ClusterData" = DataToCluster, "p" = p)

return(ClustResults)

}

# calculate the best cluster

calcTopCluster <- function(ClusteredDat, cogVars){

#Calculate the Mean of each of the cog vars for each cluster

#find the cluster that has the highest average of the means

dat<- ClusteredDat[c('Cluster', cogVars)]

s<- with(dat, aggregate(dat, by = list(Cluster), FUN = mean))

sm = c()

for(i in 1:nlevels(ClusteredDat$Cluster)){

sm[i]<- mean(t(s[i, cogVars]))

}

topCluster = levels(ClusteredDat$Cluster)[sm==max(sm)]

return(topCluster)

}

createRFModel <- function(ModelData, normalClusters){

s = 123456

clustList<-levels(ModelData$Cluster)

ClustersOfInterest<-clustList[!(clustList %in% normalClusters)]

#return(ClustResults)

MR = list() #Model Results

for (c in 1:length(ClustersOfInterest)){

ClusterofInterest<-ClustersOfInterest[c]

CI<-ClusterofInterest

MR[[CI]]$NormalData<-ModelData[ModelData$Cluster %in% normalClusters,]

MR[[CI]]$NormalData$Cluster<-'Normal'

MR[[CI]]$ClusterData<-ModelData[ModelData$Cluster == ClusterofInterest,]

MR[[CI]]$DataSubset<-rbind(MR[[CI]]$ClusterData , MR[[CI]]$NormalData)

MR[[CI]]$DataSubset$Cluster<-as.factor(as.character(MR[[CI]]$DataSubset$Cluster))

MR[[CI]]$DataSubset<-as.data.frame(MR[[CI]]$DataSubset)

set.seed(s)

inTraining<-createDataPartition(MR[[CI]]$DataSubset$Cluster, p = 0.70, list = FALSE)

MR[[CI]]$training<-MR[[CI]]$DataSubset[inTraining,]

MR[[CI]]$testing<-MR[[CI]]$DataSubset[-inTraining,]

#Smote Resampling

set.seed(s)

MR[[CI]]$smote_train <- SMOTE(Cluster ~ ., data = MR[[CI]]$DataSubset, perc.over=100)

tunegrid <- expand.grid(.mtry=c(1:ncol(ModelData)))

fitControl <- trainControl(method = "repeatedcv",

number = 10,

repeats = 5,

classProbs = TRUE,

allowParallel = TRUE,

summaryFunction = twoClassSummary

)

data = MR[[CI]]$smote_train

set.seed(s)

MR[[CI]]$FIT_RandomForest<-train( data[colnames(data)[2:length(colnames(data))]],data$Cluster, method = "rf" , ntrees = 5000, trControl = fitControl, na.action = na.pass, importance = TRUE, metric = 'ROC', seed = s)

}

#Compare Normal to All (Make This The first column of the variable importance plot)

'%!in%' <- function(x,y)!('%in%'(x,y))

CI<-'ImpairedProfile'

ClusterofInterest<-CI

MR[[CI]]$NormalData<-ModelData[ModelData$Cluster %in% normalClusters,]

MR[[CI]]$NormalData$Cluster<-'Normal'

MR[[CI]]$ClusterData<-ModelData[ModelData$Cluster %!in% normalClusters,]

MR[[CI]]$ClusterData$Cluster<-'ImpairedProfile'

MR[[CI]]$DataSubset<-rbind(MR[[CI]]$ClusterData , MR[[CI]]$NormalData)

MR[[CI]]$DataSubset$Cluster<-as.factor(as.character(MR[[CI]]$DataSubset$Cluster))

MR[[CI]]$DataSubset<-as.data.frame(MR[[CI]]$DataSubset)

set.seed(123)

inTraining<-createDataPartition(MR[[CI]]$DataSubset$Cluster, p = 0.70, list = FALSE)

MR[[CI]]$training<-MR[[CI]]$DataSubset[inTraining,]

MR[[CI]]$testing<-MR[[CI]]$DataSubset[-inTraining,]

#summary(MR[[CI]]$training)

#Smote Resampling

set.seed(1234)

MR[[CI]]$smote_train <- SMOTE(Cluster ~ ., data = MR[[CI]]$DataSubset, perc.over=100)

tunegrid <- expand.grid(.mtry=c(1:ncol(ModelData)))

fitControl <- trainControl(method = "repeatedcv",

number = 10,

repeats = 5,

classProbs = TRUE,

allowParallel = TRUE,

summaryFunction = twoClassSummary

)

data = MR[[CI]]$smote_train

set.seed(1234)

s = 1234

MR[[CI]]$FIT_RandomForest<-train( data[colnames(data)[2:length(colnames(data))]],data$Cluster, method = "rf" , ntrees = 5000, trControl = fitControl, na.action = na.pass, importance = TRUE, metric = 'ROC', seed = s)

return(MR)

}

```

<style>

.superbigimage{

overflow-x:scroll;

white-space: nowrap;

overflow-y:scroll;

max-height: 1000px;

}

.superbigimage img{

max-width: none;

</style>

# Cogntive Data Summary

```{r Load_Data, include=TRUE, warning = FALSE, echo = FALSE, fig.width = 8}

print("SOM using 10x10 grid, hexagonal topography, gaussian function. MClust 3:20")

AllData <- read_excel("C:/Users/RahaD/Dropbox/Pilot Grant/Sex Difference Profile/Sundermann UCSD Sex difference Profile ML.xlsx")

cogVars = c('tbttotc_t_pe', 'tbdtrl_t_pe','BVMTRecog_AgeEducSexRaceNormTscore', 'hpttotc_t_pe', 'hpdtrl_t_pe', 'HVLTRecog_AgeEducSexRaceNormTscore', 'mtgdtsr_n_pe', 'mtgntscr_n_pe','tratscr_n_pe','trbtscr_n_pe','ltftscr_n_pe','pc5tscr_n_pe','dytscr_n_pe')

cogVarlabels = c('BVMT Learning','BVMT Delayed Recall','BVMT Recognition','HVLT Learning','HVLT Delayed Recall','HVLT Recognition','Pegs Dominant','Pegs Non-dominant','Trails A','Trails B','FAS','PASAT 50','WAIS III Digit Symbol')

predictorVars<- c('bnviage', 'max_education', 'WRAT_last', 'WhtBlkHisOther', 'IADLComplaints', 'BDI_combined', 'LTMDD_R', 'CurrentMDD_R', 'LTorCurrAlcoholDx','LTorCurrCannabisDx' ,'LTorCurrSubsDx_NotETOHorMJ', 'Hypertension_R', 'Hyperlipidemia_R', 'Diabetes_R', 'CurrentAnticholinergicMeds_R', 'HCV_R', 'rnlog10PLASMA', 'NadirCD4', 'CD4Absolute', 'EstimatedDurationofInfectionyears', 'ARTonoff' )

factorVars<- c('WhtBlkHisOther','LTMDD_R', 'CurrentMDD_R', 'LTorCurrAlcoholDx','LTorCurrCannabisDx', 'LTorCurrSubsDx_NotETOHorMJ' ,'Hypertension_R', 'Hyperlipidemia_R', 'Diabetes_R', 'CurrentAnticholinergicMeds_R', 'HCV_R', 'ARTonoff' )

#filter data by completeness

labels(AllData[cogVars])= cogVarlabels

Data = AllData[c('linknum', cogVars)]

Data = AllData[cogVars]

#label(Data)= cogVarlabels

paste('Started with', nrow(Data), 'subjects')

AllData<- AllData[complete.cases(Data),]

labels(AllData[cogVars])= cogVarlabels

Data = AllData[cogVars]

#Plot histograms of everything - point out outliers

#Set any value<0 to 0 and any value>100 to 100

Data[Data<0]<- 0

Data[Data>100]<- 100

Data$ID = seq(1, nrow(Data))

df = melt(Data, id = "ID")

p<- ggplot(df, aes(x = value, alpha = 0.5)) + geom_density(aes(fill = variable)) + facet_wrap(~variable, scales = "free")

p<- p+ scale_fill_brewer(palette = "Paired", labels = cogVarlabels)

plot(p)

#filter by individual values

#Data <- Data%>% filter(tbttotc_t_pe>0,tbdtrl_t_pe >0, hpttotc_t_pe >0, hpdtrl_t_pe >0, mtgdtsr_n_pe >0,mtgntscr_n_pe >0, tratscr_n_pe >0,

# trbtscr_n_pe>0 , ltftscr_n_pe >0, pc5tscr_n_pe >0, BVMTRecog_AgeEducSexRaceNormTscore>-20, HVLTRecog_AgeEducSexRaceNormTscore>-20,

# dytscr_n_pe >0)

#

paste('Ended with', nrow(Data), 'subjects with complete data')

CompleteData<- AllData

CompleteData[cogVars]<- Data[cogVars]

```

## Descriptive Summaries by Gender {.tabset .tabset-fade}

### Data Table

```{r descriptive_summary_All, warning = FALSE, echo=FALSE, include=TRUE}

numVars<-predictorVars[which(!predictorVars %in% factorVars)]

catVars<- c('bngendr', factorVars)

#Make sure that All Categorical Variables are factors

ModelData<- CompleteData[c('bngendr', cogVars, predictorVars)]

ModelData[catVars] <- lapply(ModelData[catVars] , factor)

tab1<-tableby(bngendr~., data = ModelData, digits.pct = 1, digits.count = 1, numeric.stats = c("Nmiss2","meansd"), chisq.correct = TRUE, cat.test = "chisq")

tabplain<- tableby(bngendr~., data = ModelData, digits.pct = 1, digits.count = 1, numeric.stats = c("meansd"), text = TRUE)

sd<-as.data.frame(summary(tab1, text = FALSE))

sd2<-as.data.frame(summary(tab1), text = TRUE)

colnames(sd)[length(colnames(sd))]<-'p_value'

pvalscript<- sd$p_value

pvalscript[pvalscript == "< 0.001"]<- "0"

pvals<-as.numeric(pvalscript)

pvals[is.na(pvals)]<-1

colnames(sd)[1]<-'Variable'

colnames(sd2)[1]<-'Variable'

t<- tests(tab1)

options(kableExtra.auto_format = T)

#colnames(sd)[length(colnames(sd))]<-p_value

#bold the first column of anything that has a p-value

#haspvalue<- sd$`p value`!=""

sd<-as.data.table(sd)

sd%>% mutate(p_value = cell_spec(p_value , "html", background = ifelse(pvals<0.05, "yellow", "")))%>%dplyr::rename("p-value" = p_value)%>%

kable(format = "html", escape = F)%>% kable_styling(bootstrap_options = c("striped", "hover", "condensed", "responsive"))%>%

scroll_box(width = "100%", height = "500px")

```

### Continuous Data Figures

<div class="superbigimage">

```{r Continuous_Fig_All, warning = FALSE, echo=FALSE,include=TRUE, fig.height = 30, fig.width = 8}

xvar <- "bngendr"

yvars <- c(cogVars, numVars)

ModelData<-CompleteData[,c(xvar, yvars)]

tab1<- tableby(bngendr~., data = ModelData, digits.pct = 1, digits.count = 1, numeric.stats = c("meansd"))

pvals<-tests(tab1)$p

pvalscript<- paste0('p = ', round(pvals,digits = 3))

pvalscript[pvals<0.001]<- "p<0.001"

stripcolor<-ifelse(pvals<0.05, "white", "grey")

stripcolor[is.na(pvals)]<-"grey"

namedVector = setNames(pvalscript, tests(tab1)$Variable)

testn = paste(attributes(namedVector)$names, '\n',namedVector)

testn = setNames(testn, tests(tab1)$Variable)

MatchingVars <- colnames(ModelData)[2:ncol(ModelData)]

plotList <- list()

for(i in 1: length(MatchingVars)){

var = MatchingVars[i]

NewData = as.data.frame(ModelData[,c(xvar, var)])

testn <-paste(colnames(NewData)[[2]],pvalscript[i], sep = '\n' )

colnames(NewData)<-c("bngendr", "value")

NewData$bngendr<- as.factor(NewData$bngendr)

pl<-ggplot(NewData, aes(x = bngendr, y = value))

pl<- pl + geom_boxplot(width = 0.2)

pl<- pl + geom_violin(aes(fill = bngendr), draw_quantiles = .5, na.rm = TRUE, alpha = 0.5)

pl<-pl+scale_fill_brewer(palette = "Set3") + ggtitle(testn) + theme_clean()

pl<- pl+theme(text = element_text(size = 15), axis.title.y = element_blank(), axis.title.x = element_blank(),axis.text.x = element_text(angle = 45, hjust = 1), panel.background = element_rect(fill = stripcolor[i]))

#plot(pl)

plotList[[i]]<-pl

#multiplot(plotlist = plotList, cols = 4)

}

grid.arrange(grobs = plotList, ncol = 2)

```

</div>

### Categorical Data Figures

<div class="superbigimage">

```{r Grouped_Stats_Cat_Fig_All, warning = FALSE, echo=FALSE,include=TRUE, fig.height = 30, fig.width = 8}

xvar <- "bngendr"

yvars <- catVars[!catVars %in% xvar]

ModelData<-CompleteData[,c(xvar, yvars)]

ModelData<-as.data.frame(lapply(ModelData, factor))

#colnames(ModelData)<-labels(ModelData)

tab1<-tableby(as.formula(paste(xvar, "~ .")),ModelData)

pvals<-tests(tab1)$p

pvalscript<- paste0('p = ', round(pvals,digits = 3))

pvalscript[pvals<0.001]<- "p<0.001"

stripcolor<-ifelse(pvals<0.05, "white", "grey")

stripcolor[is.na(pvals)]<-"grey"

namedVector = setNames(pvalscript, tests(tab1)$Variable)

testn = paste(attributes(namedVector)$names, '\n',namedVector)

testn = setNames(testn, tests(tab1)$Variable)

MatchingVars <- colnames(ModelData)[2:ncol(ModelData)]

plotList <- list()

for(i in 1: length(MatchingVars)){

var = MatchingVars[i]

NewData = as.data.frame(ModelData[,c(paste(xvar), var)])

testn <-paste(labels(NewData)[[2]],pvalscript[i], sep = '\n' ) #paste(labels(NewData)[[2]],pvalscript[i], sep = '\n' )

##calculate percentage

#plot percentage

NewData <- NewData %>%

group_by_(xvar, var) %>%

summarise(count=n()) %>%

mutate(Percent=count/sum(count))

NewData$Percent<- NewData$Percent*100

p<-ggplot(NewData, aes_string(x =xvar, y = 'Percent')) + ggtitle(testn)

p<- p+geom_bar(stat = "identity",aes_string(fill=var))

p<- p+ scale_fill_brewer(palette = "Dark2") + theme_clean()

p<- p+ theme( text = element_text(size = 15), axis.title.y = element_blank(), axis.text.x = element_text(angle =45, hjust = 1), panel.background = element_rect(fill = stripcolor[i]))

#pl

plotList[[i]] <- p

# HTMLplot(GraphRes = 500)

}

grid.arrange(grobs = plotList, ncol = 2)

```

</div>

## Cluster Results

```{r Cluster_All, warning = FALSE, echo=FALSE,include=TRUE, fig.width = 8}

ClusterData_All<- CompleteData

DataInfo = 'All Participants'

AllClusteredList<- createAndPlotClustersSOM(ClusterData_All, cogVars, DataInfo)

p<- AllClusteredList$p

p<- p+ scale_fill_brewer(palette = "Paired", labels = cogVarlabels)

plot(p)

```

## Cluster Statistics{.tabset .tabset-fade}

### Data Table by Cluster

```{r Table_All, warning = FALSE, echo=FALSE, include=TRUE}

numVars<-predictorVars[which(!predictorVars %in% factorVars)]

catVars<- c('Cluster', 'bngendr', factorVars)

#Make sure that All Categorical Variables are factors

ModelData<-AllClusteredList$ClusterData[c('Cluster', 'bngendr', cogVars, predictorVars)]

ModelData[catVars] <- lapply(ModelData[catVars] , factor)

tab1<-tableby(Cluster~., data = ModelData, digits.pct = 1, digits.count = 1, numeric.stats = c("Nmiss2", "meansd"), cat.test = "chisq")

tabplain<- tableby(Cluster~., data = ModelData, digits.pct = 1, digits.count = 1, numeric.stats = c("meansd"), text = TRUE)

sd<-as.data.frame(summary(tab1, text = FALSE))

sd2<-as.data.frame(summary(tab1), text = TRUE)

colnames(sd)[length(colnames(sd))]<-'p_value'

pvalscript<- sd$p_value

pvalscript[pvalscript == "< 0.001"]<- "0"

pvals<-as.numeric(pvalscript)

pvals[is.na(pvals)]<-1

colnames(sd)[1]<-'Variable'

colnames(sd2)[1]<-'Variable'

t<- tests(tab1)

options(kableExtra.auto_format = T)

#colnames(sd)[length(colnames(sd))]<-p_value

#bold the first column of anything that has a p-value

#haspvalue<- sd$`p value`!=""

sd<-as.data.table(sd)

sd%>% mutate(p_value = cell_spec(p_value , "html", background = ifelse(pvals<0.05, "yellow", "")))%>%dplyr::rename("p-value" = p_value)%>%

kable(format = "html", escape = F)%>% kable_styling(bootstrap_options = c("striped", "hover", "condensed", "responsive"))%>%

scroll_box(width = "100%", height = "500px")

```

<div class="tocify-extend-page" data-unique="tocify-extend-page" style="height: 0;"></div>

### Continuous Data Figures

<div class="superbigimage">

```{r Continuous_Fig_ClustAll, warning = FALSE, echo=FALSE,include=TRUE, fig.height = 30, fig.width = 8}

xvar <- "Cluster"

yvars <- c(cogVars, numVars)

ModelData<-AllClusteredList$ClusterData[,c(xvar, yvars)]

tab1<- tableby(Cluster~., data = ModelData, digits.pct = 1, digits.count = 1, numeric.stats = c("meansd"))

pvals<-tests(tab1)$p

pvalscript<- paste0('p = ', round(pvals,digits = 3))

pvalscript[pvals<0.001]<- "p<0.001"

stripcolor<-ifelse(pvals<0.05, "white", "grey")

stripcolor[is.na(pvals)]<-"grey"

namedVector = setNames(pvalscript, tests(tab1)$Variable)

testn = paste(attributes(namedVector)$names, '\n',namedVector)

testn = setNames(testn, tests(tab1)$Variable)

MatchingVars <- colnames(ModelData)[2:ncol(ModelData)]

plotList <- list()

for(i in 1: length(MatchingVars)){

var = MatchingVars[i]

NewData = as.data.frame(ModelData[,c(xvar, var)])

testn <-paste(colnames(NewData)[[2]],pvalscript[i], sep = '\n' )

colnames(NewData)<-c("Cluster", "value")

NewData$Cluster<- as.factor(NewData$Cluster)

pl<-ggplot(NewData, aes(x = Cluster, y = value))

pl<- pl + geom_boxplot(width = 0.2)

pl<- pl + geom_violin(aes(fill = Cluster), draw_quantiles = .5, na.rm = TRUE, alpha = 0.5)

pl<-pl+scale_fill_brewer(palette = "Set3") + ggtitle(testn) + theme_clean()

pl<- pl+theme(text = element_text(size = 15), axis.title.y = element_blank(), axis.title.x = element_blank(),axis.text.x = element_text(angle = 45, hjust = 1), panel.background = element_rect(fill = stripcolor[i]))

#plot(pl)

plotList[[i]]<-pl

#multiplot(plotlist = plotList, cols = 4)

}

grid.arrange(grobs = plotList, ncol = 2)

```

</div>

### Categorical Data Figures

<div class="superbigimage">

```{r Cat_Fig_All, warning = FALSE, echo=FALSE,include=TRUE, fig.height = 30, fig.width = 8}

xvar <- "Cluster"

yvars <- catVars[!catVars %in% xvar]

ModelData<-AllClusteredList$ClusterData[,c(xvar, 'bngendr',yvars)]

ModelData<-as.data.frame(lapply(ModelData, factor))

#colnames(ModelData)<-labels(ModelData)

tab1<-tableby(as.formula(paste(xvar, "~ .")),ModelData)

pvals<-tests(tab1)$p

pvalscript<- paste0('p = ', round(pvals,digits = 3))

pvalscript[pvals<0.001]<- "p<0.001"

stripcolor<-ifelse(pvals<0.05, "white", "grey")

stripcolor[is.na(pvals)]<-"grey"

namedVector = setNames(pvalscript, tests(tab1)$Variable)

testn = paste(attributes(namedVector)$names, '\n',namedVector)

testn = setNames(testn, tests(tab1)$Variable)

MatchingVars <- colnames(ModelData)[2:ncol(ModelData)]

plotList <- list()

for(i in 1: length(MatchingVars)){

var = MatchingVars[i]

NewData = as.data.frame(ModelData[,c(paste(xvar), var)])

testn <-paste(labels(NewData)[[2]],pvalscript[i], sep = '\n' ) #paste(labels(NewData)[[2]],pvalscript[i], sep = '\n' )

##calculate percentage

#plot percentage

NewData <- NewData %>%

group_by_(xvar, var) %>%

summarise(count=n()) %>%

mutate(Percent=count/sum(count))

NewData$Percent<- NewData$Percent*100

p<-ggplot(NewData, aes_string(x =xvar, y = 'Percent')) + ggtitle(testn)

p<- p+geom_bar(stat = "identity",aes_string(fill=var))

p<- p+ scale_fill_brewer(palette = "Dark2") + theme_clean()

p<- p+ theme( text = element_text(size = 15), axis.title.y = element_blank(), axis.text.x = element_text(angle =45, hjust = 1), panel.background = element_rect(fill = stripcolor[i]))

#pl

plotList[[i]] <- p

# HTMLplot(GraphRes = 500)

}

grid.arrange(grobs = plotList, ncol = 2)

```

</div>

## Random Forest Model Creation: All Data

```{r RF_All, warning = FALSE, echo=FALSE,include=TRUE}

#figure out which clusters are normal and which arent

ModelData_All<- AllClusteredList$ClusterData

catVars<- c('Cluster', 'bngendr', factorVars)

#Make sure that All Categorical Variables are factors

ModelData_All[catVars]<- lapply(ModelData_All[catVars],as.character)

ModelData_All[catVars] <- lapply(ModelData_All[catVars] , factor)

ModelData_All[catVars]<- lapply(ModelData_All[catVars], make.names)

#if there's a dot, remove it

ModelData<- as.data.frame(ModelData_All[c('Cluster', 'bngendr', predictorVars)])

ModelData[catVars][ModelData[catVars]=="NA."] <- NA

ModelData[catVars] <- lapply(ModelData[catVars] , factor)

ModelData_All[catVars] <- lapply(ModelData_All[catVars] , factor)

topClust = calcTopCluster(ModelData_All, cogVars)

paste("The normal cluster is detected as being:", topClust)

#Remove Variables before runing through RF

all_f = sapply(ModelData[,catVars], function(x) nlevels(x)>1)

removed_onelevelfactors <- names(all_f[all_f==FALSE])

paste('The following Variables were removed because they had less than 2 factors: ', toString(removed_onelevelfactors))

ModelData<-select(ModelData, -removed_onelevelfactors)

#No go through and look for missing variables

aggr_plot_all <- aggr(ModelData, col=c('navyblue','red'), numbers=TRUE, sortVars=TRUE,cex.axis=.7, gap=3, ylab=c("Histogram of missing data","Pattern"), bars = TRUE, labels = TRUE, plot = FALSE)

thresh = .5 * nrow(ModelData)

remove_ind = aggr_plot_all$missings$Count>thresh

removed_30pmissing<-colnames(ModelData[,remove_ind])

paste('The following Variables were removed because they had more than 50% missing: ', toString(removed_30pmissing))

ModelData<-select(ModelData, -removed_30pmissing)

lowVarPredictors = nearZeroVar(ModelData, saveMetrics = TRUE)

removed_lowVar<- rownames(lowVarPredictors)[lowVarPredictors$nzv==TRUE ]

paste('The following Variables were removed because they had near zero variance: ', toString(rownames(lowVarPredictors)[lowVarPredictors$nzv==TRUE ]))

ModelData<-select(ModelData, rownames(lowVarPredictors)[lowVarPredictors$nzv==FALSE])

paste('Final Variables: ', toString(colnames(ModelData)))

#impute the other variables

imputed_temp <-mice(ModelData, meth = "rf", seed = 123, printFlag = FALSE)

ModelData_imputed_all = mice::complete(imputed_temp)

RF_all<- createRFModel(ModelData_imputed_all, topClust)

remove('df', 'imputed_temp', 'lowVarPredictors', 'ModelData',' NewData', 'p', 'pl', 'plotList', 'sd', 'sd2', 't', 'tab1', 'tabplain', 'i', 'remove_ind', 'remove_30pmissing', 'stripcolor', 'testn', 'thresh', 'var', 'topClust')

```

## All: Random Forest Results {.tabset .tabset-fade}

### Variable Importance

```{r All_RF_VI, warning = FALSE, echo=FALSE, include=TRUE}

##Calculate and Plot Variable Importance Using different sorting methods

MR<- RF_all

groups<- attributes(MR)$names

CIs<- groups[1:length(groups)-1]

if(length(CIs)==1){

CIs <- "ImpairedProfile"

}else{

CIs<-c('ImpairedProfile',CIs)

}

for (c in 1:length(CIs)){

ClusterofInterest<-CIs[c]

CI<-ClusterofInterest

Model_RF <- MR[[CI]]$FIT_RandomForest

model = Model_RF

model_label<- CI

imps = varImp(model, scale = TRUE)

if(c==1){

Imp_df = data.frame(row.names = predictors(model))

Imp_df$ID <-seq.int(nrow(Imp_df))

Imp_df$Variable <-rownames(Imp_df)

Imp_df$Variable<-factor(Imp_df$Variable, levels = rev(Imp_df$Variable) )

}

imps2 = imps[["importance"]]

if (ncol(imps2) ==2){

imps2 = imps2[ClusterofInterest]

}

colnames(imps2) = model_label

imps2$Variable = rownames(imps2)

Imp_df<-merge(Imp_df, imps2,by = "Variable", all = TRUE )

}

newdf<-Imp_df[order(Imp_df$ImpairedProfile),]

newdf$ID <-seq.int(nrow(newdf))

#newdf$Variable<- VarTypes1[[2]][match(newdf$Variable, VarTypes1[[1]])]

dfImp<-melt(newdf, id = c('ID', 'Variable') )

#Rename Variables

##Try highlighing top value

me.2 <- dfImp %>%

group_by(variable) %>% mutate(color = value %in% head(sort(value, decreasing = TRUE),10) )

p<- ggplot(me.2, aes(x = Variable, y = value)) +coord_flip()

p<- p + geom_segment(size = 2,aes(x = Variable, xend = Variable, y =0, yend = value, color = color )) + scale_x_discrete(limits = newdf$Variable)

p<-p + facet_wrap(~variable, nrow=1) +theme(legend.position = "none", strip.text.y = element_text(angle=45), axis.title.x = element_blank(), axis.title.y = element_blank())

p<- p + geom_point() +ylab('Variable Importance') + ggtitle('Random Forest Variable Importance') +scale_color_manual(values = c("lightgrey", "black"))

plot(p)

```

### ROC Plots

```{r All_RF_ROC, warning = FALSE, echo=FALSE, include=TRUE}

##Plot ROC

for (c in 1:length(CIs)){

CI<-CIs[c]

#Plot ROCs

model <- MR[[CI]]$FIT_RandomForest

Pred_RF <-predict(model, MR[[CI]]$DataSubset, type = "prob")

ROC_obj<-roc(response = MR[[CI]]$DataSubset$Cluster, predictor = Pred_RF[,CI], ci = TRUE)

ci.sp.obj <- ci.sp(ROC_obj, sensitivities=seq(0, 1, .01))

plot(ROC_obj)

plot(ci.sp.obj, type="shape", col="lightblue")

title(CI, line = 2)

print(model)

print(model$finalModel)

}

remove('dfImp', 'Imp_df', 'imps', 'imps2', 'me.2', 'model', 'model_RF', 'modelData_imputed', 'MR', 'NewData', 'newdf', 'Pred_RF', 'RF', 'ROC_obj', 'all_f', 'c', 'CI', 'CIs', 'groups', 'MatchingVars', 'model_label', 'namedVector', 'pvalscript', 'removed_30pmissing', 'removed_lowVar', 'removed_onelevelfactors', 'p' )

```

# Males Only

## Cluster Results

```{r Cluster_Men, warning = FALSE, echo=FALSE,include=TRUE, fig.width = 8}

ClusterData_Men<- CompleteData[CompleteData$bngendr==1,]

DataInfo = 'Males'

MenClusteredList<- createAndPlotClustersSOM(ClusterData_Men, cogVars, DataInfo)

p<- MenClusteredList$p

p<- p+ scale_fill_brewer(palette = "Paired", labels = cogVarlabels)

plot(p)

```

## Cluster Statistics{.tabset .tabset-fade}

### Data Table by Cluster

```{r Table_Men, warning = FALSE, echo=FALSE, include=TRUE}

numVars<-predictorVars[which(!predictorVars %in% factorVars)]

catVars<- c('Cluster', factorVars)

#Make sure that All Categorical Variables are factors

ModelData<-MenClusteredList$ClusterData[c('Cluster', cogVars, predictorVars)]

ModelData[catVars] <- lapply(ModelData[catVars] , factor)

tab1<-tableby(Cluster~., data = ModelData, digits.pct = 1, digits.count = 1, numeric.stats = c("Nmiss2", "meansd"), cat.test = "chisq")

tabplain<- tableby(Cluster~., data = ModelData, digits.pct = 1, digits.count = 1, numeric.stats = c("meansd"), text = TRUE)

sd<-as.data.frame(summary(tab1, text = FALSE))

sd2<-as.data.frame(summary(tab1), text = TRUE)

colnames(sd)[length(colnames(sd))]<-'p_value'

pvalscript<- sd$p_value

pvalscript[pvalscript == "< 0.001"]<- "0"

pvals<-as.numeric(pvalscript)

pvals[is.na(pvals)]<-1

colnames(sd)[1]<-'Variable'

colnames(sd2)[1]<-'Variable'

t<- tests(tab1)

options(kableExtra.auto_format = T)

#colnames(sd)[length(colnames(sd))]<-p_value

#bold the first column of anything that has a p-value

#haspvalue<- sd$`p value`!=""

sd<-as.data.table(sd)

sd%>% mutate(p_value = cell_spec(p_value , "html", background = ifelse(pvals<0.05, "yellow", "")))%>%dplyr::rename("p-value" = p_value)%>%

kable(format = "html", escape = F)%>% kable_styling(bootstrap_options = c("striped", "hover", "condensed", "responsive"))%>%

scroll_box(width = "100%", height = "500px")

```

### Continuous Data Figures

<div class="superbigimage">

```{r Continuous_Fig_ClustMen, warning = FALSE, echo=FALSE,include=TRUE, fig.height = 30, fig.width = 8}

xvar <- "Cluster"

yvars <- c(cogVars, numVars)

ModelData<-MenClusteredList$ClusterData[,c(xvar, yvars)]

tab1<- tableby(Cluster~., data = ModelData, digits.pct = 1, digits.count = 1, numeric.stats = c("meansd"))

pvals<-tests(tab1)$p

pvalscript<- paste0('p = ', round(pvals,digits = 3))

pvalscript[pvals<0.001]<- "p<0.001"

stripcolor<-ifelse(pvals<0.05, "white", "grey")

stripcolor[is.na(pvals)]<-"grey"

namedVector = setNames(pvalscript, tests(tab1)$Variable)

testn = paste(attributes(namedVector)$names, '\n',namedVector)

testn = setNames(testn, tests(tab1)$Variable)

MatchingVars <- colnames(ModelData)[2:ncol(ModelData)]

plotList <- list()

for(i in 1: length(MatchingVars)){

var = MatchingVars[i]

NewData = as.data.frame(ModelData[,c(xvar, var)])

testn <-paste(colnames(NewData)[[2]],pvalscript[i], sep = '\n' )

colnames(NewData)<-c("Cluster", "value")

NewData$Cluster<- as.factor(NewData$Cluster)

pl<-ggplot(NewData, aes(x = Cluster, y = value))

pl<- pl + geom_boxplot(width = 0.2)

pl<- pl + geom_violin(aes(fill = Cluster), draw_quantiles = .5, na.rm = TRUE, alpha = 0.5)

pl<-pl+scale_fill_brewer(palette = "Set3") + ggtitle(testn) + theme_clean()

pl<- pl+theme(text = element_text(size = 15), axis.title.y = element_blank(), axis.title.x = element_blank(),axis.text.x = element_text(angle = 45, hjust = 1), panel.background = element_rect(fill = stripcolor[i]))

#plot(pl)

plotList[[i]]<-pl

#multiplot(plotlist = plotList, cols = 4)

}

grid.arrange(grobs = plotList, ncol = 2)

```

</div>

### Categorical Data Figures

<div class="superbigimage">

```{r Cat_Fig_Men, warning = FALSE, echo=FALSE,include=TRUE, fig.height = 30, fig.width = 8}

xvar <- "Cluster"

yvars <- catVars[!catVars %in% xvar]

ModelData<-MenClusteredList$ClusterData[,c(xvar, yvars)]

ModelData<-as.data.frame(lapply(ModelData, factor))

#colnames(ModelData)<-labels(ModelData)

tab1<-tableby(as.formula(paste(xvar, "~ .")),ModelData)

pvals<-tests(tab1)$p

pvalscript<- paste0('p = ', round(pvals,digits = 3))

pvalscript[pvals<0.001]<- "p<0.001"

stripcolor<-ifelse(pvals<0.05, "white", "grey")

stripcolor[is.na(pvals)]<-"grey"

namedVector = setNames(pvalscript, tests(tab1)$Variable)

testn = paste(attributes(namedVector)$names, '\n',namedVector)

testn = setNames(testn, tests(tab1)$Variable)

MatchingVars <- colnames(ModelData)[2:ncol(ModelData)]

plotList <- list()

for(i in 1: length(MatchingVars)){

var = MatchingVars[i]

NewData = as.data.frame(ModelData[,c(paste(xvar), var)])

testn <-paste(labels(NewData)[[2]],pvalscript[i], sep = '\n' ) #paste(labels(NewData)[[2]],pvalscript[i], sep = '\n' )

##calculate percentage

#plot percentage

NewData <- NewData %>%

group_by_(xvar, var) %>%

summarise(count=n()) %>%

mutate(Percent=count/sum(count))

NewData$Percent<- NewData$Percent*100

p<-ggplot(NewData, aes_string(x =xvar, y = 'Percent')) + ggtitle(testn)

p<- p+geom_bar(stat = "identity",aes_string(fill=var))

p<- p+ scale_fill_brewer(palette = "Dark2") + theme_clean()

p<- p+ theme( text = element_text(size = 15), axis.title.y = element_blank(), axis.text.x = element_text(angle =45, hjust = 1), panel.background = element_rect(fill = stripcolor[i]))

#pl

plotList[[i]] <- p

# HTMLplot(GraphRes = 500)

}

grid.arrange(grobs = plotList, ncol = 2)

```

</div>

## Random Forest Model Creation: Males

```{r RF_Men, warning = FALSE, echo=FALSE,include=TRUE}

#figure out which clusters are normal and which arent

ModelData_Men<- MenClusteredList$ClusterData

catVars<- c('Cluster', factorVars)

#Make sure that All Categorical Variables are factors

ModelData_Men[catVars]<- lapply(ModelData_Men[catVars],as.character)

ModelData_Men[catVars] <- lapply(ModelData_Men[catVars] , factor)

ModelData_Men[catVars]<- lapply(ModelData_Men[catVars], make.names)

#if there's a dot, remove it

ModelData<- as.data.frame(ModelData_Men[c('Cluster', predictorVars)])

ModelData[catVars][ModelData[catVars]=="NA."] <- NA

ModelData[catVars] <- lapply(ModelData[catVars] , factor)

ModelData_Men[catVars] <- lapply(ModelData_Men[catVars] , factor)

topClust = calcTopCluster(ModelData_Men, cogVars)

paste("The normal cluster is detected as being:", topClust)

#Remove Variables before runing through RF

all_f = sapply(ModelData[,catVars], function(x) nlevels(x)>1)

removed_onelevelfactors <- names(all_f[all_f==FALSE])

paste('The following Variables were removed because they had less than 2 factors: ', toString(removed_onelevelfactors))

ModelData<-select(ModelData, -removed_onelevelfactors)

#No go through and look for missing variables

aggr_plot_all <- aggr(ModelData, col=c('navyblue','red'), numbers=TRUE, sortVars=TRUE,cex.axis=.7, gap=3, ylab=c("Histogram of missing data","Pattern"), bars = TRUE, labels = TRUE, plot = FALSE)

thresh = .5 * nrow(ModelData)

remove_ind = aggr_plot_all$missings$Count>thresh

removed_30pmissing<-colnames(ModelData[,remove_ind])

paste('The following Variables were removed because they had more than 50% missing: ', toString(removed_30pmissing))

ModelData<-select(ModelData, -removed_30pmissing)

lowVarPredictors = nearZeroVar(ModelData, saveMetrics = TRUE)

removed_lowVar<- rownames(lowVarPredictors)[lowVarPredictors$nzv==TRUE ]

paste('The following Variables were removed because they had near zero variance: ', toString(rownames(lowVarPredictors)[lowVarPredictors$nzv==TRUE ]))

ModelData<-select(ModelData, rownames(lowVarPredictors)[lowVarPredictors$nzv==FALSE])

paste('Final Variables: ', toString(colnames(ModelData)))

#impute the other variables

imputed_temp <-mice(ModelData, meth = "rf", seed = 123, printFlag = FALSE)

ModelData_imputed_Men = mice::complete(imputed_temp)

RF_Men<- createRFModel(ModelData_imputed_Men, topClust)

remove('df', 'imputed_temp', 'lowVarPredictors', 'ModelData',' NewData', 'p', 'pl', 'plotList', 'sd', 'sd2', 't', 'tab1', 'tabplain', 'i', 'remove_ind', 'remove_30pmissing', 'stripcolor', 'testn', 'thresh', 'var', 'topClust')

```

## Males: Random Forest Results {.tabset .tabset-fade}

### Variable Importance

```{r Men_RF_VI, warning = FALSE, echo=FALSE, include=TRUE}

##Calculate and Plot Variable Importance Using different sorting methods

MR<- RF_Men

groups<- attributes(MR)$names

CIs<- groups[1:length(groups)-1]

if(length(CIs)==1){

CIs <- "ImpairedProfile"

}else{

CIs<-c('ImpairedProfile',CIs)

}

for (c in 1:length(CIs)){

ClusterofInterest<-CIs[c]

CI<-ClusterofInterest

Model_RF <- MR[[CI]]$FIT_RandomForest

model = Model_RF

model_label<- CI

imps = varImp(model, scale = TRUE)

if(c==1){

Imp_df = data.frame(row.names = predictors(model))

Imp_df$ID <-seq.int(nrow(Imp_df))

Imp_df$Variable <-rownames(Imp_df)

Imp_df$Variable<-factor(Imp_df$Variable, levels = rev(Imp_df$Variable) )

}

imps2 = imps[["importance"]]

if (ncol(imps2) ==2){

imps2 = imps2[ClusterofInterest]

}

colnames(imps2) = model_label

imps2$Variable = rownames(imps2)

Imp_df<-merge(Imp_df, imps2,by = "Variable", all = TRUE )

}

newdf<-Imp_df[order(Imp_df$ImpairedProfile),]

newdf$ID <-seq.int(nrow(newdf))

#newdf$Variable<- VarTypes1[[2]][match(newdf$Variable, VarTypes1[[1]])]

dfImp<-melt(newdf, id = c('ID', 'Variable') )

#Rename Variables

##Try highlighing top value

me.2 <- dfImp %>%

group_by(variable) %>% mutate(color = value %in% head(sort(value, decreasing = TRUE),10) )

p<- ggplot(me.2, aes(x = Variable, y = value)) +coord_flip()

p<- p + geom_segment(size = 2,aes(x = Variable, xend = Variable, y =0, yend = value, color = color )) + scale_x_discrete(limits = newdf$Variable)

p<-p + facet_wrap(~variable, nrow=1) +theme(legend.position = "none", strip.text.y = element_text(angle=45), axis.title.x = element_blank(), axis.title.y = element_blank())

p<- p + geom_point() +ylab('Variable Importance') + ggtitle('Random Forest Variable Importance') +scale_color_manual(values = c("lightgrey", "black"))

plot(p)

```

### ROC Plots

```{r Men_RF_ROC, warning = FALSE, echo=FALSE, include=TRUE}

##Plot ROC

for (c in 1:length(CIs)){

CI<-CIs[c]

#Plot ROCs

model <- MR[[CI]]$FIT_RandomForest

Pred_RF <-predict(model, MR[[CI]]$DataSubset, type = "prob")

ROC_obj<-roc(response = MR[[CI]]$DataSubset$Cluster, predictor = Pred_RF[,CI], ci = TRUE)

ci.sp.obj <- ci.sp(ROC_obj, sensitivities=seq(0, 1, .01))

plot(ROC_obj)

plot(ci.sp.obj, type="shape", col="lightblue")

title(CI, line = 2)

print(model)

print(model$finalModel)

}

remove('dfImp', 'Imp_df', 'imps', 'imps2', 'me.2', 'model', 'model_RF', 'modelData_imputed', 'MR', 'NewData', 'newdf', 'Pred_RF', 'RF', 'ROC_obj', 'all_f', 'c', 'CI', 'CIs', 'groups', 'MatchingVars', 'model_label', 'namedVector', 'pvalscript', 'removed_30pmissing', 'removed_lowVar', 'removed_onelevelfactors', 'p' )

```

# Females Only

## Cluster Results

```{r Cluster_Women, warning = FALSE, echo=FALSE,include=TRUE, fig.width = 8}

ClusterData_Women<- CompleteData[CompleteData$bngendr==2,]

DataInfo = 'Females'

WomenClusteredList<- createAndPlotClustersSOM(ClusterData_Women, cogVars, DataInfo)

p<- WomenClusteredList$p

p<- p+ scale_fill_brewer(palette = "Paired", labels = cogVarlabels)

plot(p)

```

## Cluster Statistics{.tabset .tabset-fade}

### Data Table by Cluster

```{r Table_Women, warning = FALSE, echo=FALSE, include=TRUE}

numVars<-predictorVars[which(!predictorVars %in% factorVars)]

catVars<- c('Cluster', factorVars)

#Make sure that All Categorical Variables are factors

ModelData<-WomenClusteredList$ClusterData[c('Cluster', cogVars, predictorVars)]

ModelData[catVars] <- lapply(ModelData[catVars] , factor)

tab1<-tableby(Cluster~., data = ModelData, digits.pct = 1, digits.count = 1, numeric.stats = c("Nmiss2", "meansd"), cat.test = "chisq")

tabplain<- tableby(Cluster~., data = ModelData, digits.pct = 1, digits.count = 1, numeric.stats = c("meansd"), text = TRUE)

sd<-as.data.frame(summary(tab1, text = FALSE))

sd2<-as.data.frame(summary(tab1), text = TRUE)

colnames(sd)[length(colnames(sd))]<-'p_value'

pvalscript<- sd$p_value

pvalscript[pvalscript == "< 0.001"]<- "0"

pvals<-as.numeric(pvalscript)

pvals[is.na(pvals)]<-1

colnames(sd)[1]<-'Variable'

colnames(sd2)[1]<-'Variable'

t<- tests(tab1)

options(kableExtra.auto_format = T)

#colnames(sd)[length(colnames(sd))]<-p_value

#bold the first column of anything that has a p-value

#haspvalue<- sd$`p value`!=""

sd<-as.data.table(sd)

sd%>% mutate(p_value = cell_spec(p_value , "html", background = ifelse(pvals<0.05, "yellow", "")))%>%dplyr::rename("p-value" = p_value)%>%

kable(format = "html", escape = F)%>% kable_styling(bootstrap_options = c("striped", "hover", "condensed", "responsive"))%>%

scroll_box(width = "100%", height = "500px")

```

### Continuous Data Figures

<div class="superbigimage">

```{r Continuous_Fig_ClustWomen, warning = FALSE, echo=FALSE,include=TRUE, fig.height = 30, fig.width = 8}

xvar <- "Cluster"

yvars <- c(cogVars, numVars)

ModelData<-WomenClusteredList$ClusterData[,c(xvar, yvars)]

tab1<- tableby(Cluster~., data = ModelData, digits.pct = 1, digits.count = 1, numeric.stats = c("meansd"))

pvals<-tests(tab1)$p

pvalscript<- paste0('p = ', round(pvals,digits = 3))

pvalscript[pvals<0.001]<- "p<0.001"

stripcolor<-ifelse(pvals<0.05, "white", "grey")

stripcolor[is.na(pvals)]<-"grey"

namedVector = setNames(pvalscript, tests(tab1)$Variable)

testn = paste(attributes(namedVector)$names, '\n',namedVector)

testn = setNames(testn, tests(tab1)$Variable)

MatchingVars <- colnames(ModelData)[2:ncol(ModelData)]

plotList <- list()

for(i in 1: length(MatchingVars)){

var = MatchingVars[i]

NewData = as.data.frame(ModelData[,c(xvar, var)])

testn <-paste(colnames(NewData)[[2]],pvalscript[i], sep = '\n' )

colnames(NewData)<-c("Cluster", "value")

NewData$Cluster<- as.factor(NewData$Cluster)

pl<-ggplot(NewData, aes(x = Cluster, y = value))

pl<- pl + geom_boxplot(width = 0.2)

pl<- pl + geom_violin(aes(fill = Cluster), draw_quantiles = .5, na.rm = TRUE, alpha = 0.5)

pl<-pl+scale_fill_brewer(palette = "Set3") + ggtitle(testn) + theme_clean()

pl<- pl+theme(text = element_text(size = 15), axis.title.y = element_blank(), axis.title.x = element_blank(),axis.text.x = element_text(angle = 45, hjust = 1), panel.background = element_rect(fill = stripcolor[i]))

#plot(pl)

plotList[[i]]<-pl

#multiplot(plotlist = plotList, cols = 4)

}

grid.arrange(grobs = plotList, ncol = 2)

```

</div>

### Categorical Data Figures

<div class="superbigimage">

```{r Cat_Fig_Women, warning = FALSE, echo=FALSE,include=TRUE, fig.height = 30, fig.width = 8}

xvar <- "Cluster"

yvars <- catVars[!catVars %in% xvar]

ModelData<-WomenClusteredList$ClusterData[,c(xvar, yvars)]

ModelData<-as.data.frame(lapply(ModelData, factor))

#colnames(ModelData)<-labels(ModelData)

tab1<-tableby(as.formula(paste(xvar, "~ .")),ModelData)

pvals<-tests(tab1)$p

pvalscript<- paste0('p = ', round(pvals,digits = 3))

pvalscript[pvals<0.001]<- "p<0.001"

stripcolor<-ifelse(pvals<0.05, "white", "grey")

stripcolor[is.na(pvals)]<-"grey"

namedVector = setNames(pvalscript, tests(tab1)$Variable)

testn = paste(attributes(namedVector)$names, '\n',namedVector)

testn = setNames(testn, tests(tab1)$Variable)

MatchingVars <- colnames(ModelData)[2:ncol(ModelData)]

plotList <- list()

for(i in 1: length(MatchingVars)){

var = MatchingVars[i]

NewData = as.data.frame(ModelData[,c(paste(xvar), var)])

testn <-paste(labels(NewData)[[2]],pvalscript[i], sep = '\n' ) #paste(labels(NewData)[[2]],pvalscript[i], sep = '\n' )

##calculate percentage

#plot percentage

NewData <- as.data.frame(NewData) %>%

group_by_(xvar, var) %>%

dplyr::summarise(count=n()) %>%

mutate(Percent=count/sum(count))

NewData$Percent<- NewData$Percent*100

p<-ggplot(NewData, aes_string(x =xvar, y = 'Percent')) + ggtitle(testn)

p<- p+geom_bar(stat = "identity",aes_string(fill=var))

p<- p+ scale_fill_brewer(palette = "Dark2") + theme_clean()

p<- p+ theme( text = element_text(size = 15), axis.title.y = element_blank(), axis.text.x = element_text(angle =45, hjust = 1), panel.background = element_rect(fill = stripcolor[i]))

#pl

plotList[[i]] <- p

# HTMLplot(GraphRes = 500)

}

grid.arrange(grobs = plotList, ncol = 2)

```

</div>

## Random Forest Model Creation: Females

```{r RF_Women, warning = FALSE, echo=FALSE,include=TRUE}

#figure out which clusters are normal and which arent

ModelData_Women<- WomenClusteredList$ClusterData

catVars<- c('Cluster', factorVars)

#Make sure that All Categorical Variables are factors

ModelData_Women[catVars]<- lapply(ModelData_Women[catVars],as.character)

ModelData_Women[catVars] <- lapply(ModelData_Women[catVars] , factor)

ModelData_Women[catVars]<- lapply(ModelData_Women[catVars], make.names)

#if there's a dot, remove it

ModelData<- as.data.frame(ModelData_Women[c('Cluster', predictorVars)])

ModelData[catVars][ModelData[catVars]=="NA."] <- NA

ModelData[catVars] <- lapply(ModelData[catVars] , factor)

ModelData_Women[catVars] <- lapply(ModelData_Women[catVars] , factor)

topClust = calcTopCluster(ModelData_Women, cogVars)

paste("The normal cluster is detected as being:", topClust)

#Remove Variables before runing through RF

all_f = sapply(ModelData[,catVars], function(x) nlevels(x)>1)

removed_onelevelfactors <- names(all_f[all_f==FALSE])

paste('The following Variables were removed because they had less than 2 factors: ', toString(removed_onelevelfactors))

ModelData<-select(ModelData, -removed_onelevelfactors)

#Now go through and look for missing variables

aggr_plot_all <- aggr(ModelData, col=c('navyblue','red'), numbers=TRUE, sortVars=TRUE,cex.axis=.7, gap=3, ylab=c("Histogram of missing data","Pattern"), bars = TRUE, labels = TRUE, plot = FALSE)

thresh = .5 * nrow(ModelData)

remove_ind = aggr_plot_all$missings$Count>thresh

removed_30pmissing<-colnames(ModelData[,remove_ind])

paste('The following Variables were removed because they had more than 50% missing: ', toString(removed_30pmissing))

ModelData<-select(ModelData, -removed_30pmissing)

lowVarPredictors = nearZeroVar(ModelData, saveMetrics = TRUE)

removed_lowVar<- rownames(lowVarPredictors)[lowVarPredictors$nzv==TRUE ]

paste('The following Variables were removed because they had near zero variance: ', toString(rownames(lowVarPredictors)[lowVarPredictors$nzv==TRUE ]))

ModelData<-select(ModelData, rownames(lowVarPredictors)[lowVarPredictors$nzv==FALSE])

paste('Final Variables: ', toString(colnames(ModelData)))

#impute the other variables

imputed_temp <-mice(ModelData, meth = "rf", seed = 123, printFlag = FALSE)

ModelData_imputed_Women = mice::complete(imputed_temp)

RF_Women<- createRFModel(ModelData_imputed_Women, topClust)

remove('df', 'imputed_temp', 'lowVarPredictors', 'ModelData',' NewData', 'p', 'pl', 'plotList', 'sd', 'sd2', 't', 'tab1', 'tabplain', 'i', 'remove_ind', 'remove_30pmissing', 'stripcolor', 'testn', 'thresh', 'var', 'topClust')

```

## Females: Random Forest Results {.tabset .tabset-fade}

### Variable Importance

```{r Women_RF_VI, warning = FALSE, echo=FALSE, include=TRUE}

##Calculate and Plot Variable Importance Using different sorting methods

MR<- RF_Women

groups<- attributes(MR)$names

CIs<- groups[1:length(groups)-1]

if(length(CIs)==1){

CIs <- "ImpairedProfile"

}else{

CIs<-c('ImpairedProfile',CIs)

}

for (c in 1:length(CIs)){

ClusterofInterest<-CIs[c]

CI<-ClusterofInterest

Model_RF <- MR[[CI]]$FIT_RandomForest

model = Model_RF

model_label<- CI

imps = varImp(model, scale = TRUE)

if(c==1){

Imp_df = data.frame(row.names = predictors(model))

Imp_df$ID <-seq.int(nrow(Imp_df))

Imp_df$Variable <-rownames(Imp_df)

Imp_df$Variable<-factor(Imp_df$Variable, levels = rev(Imp_df$Variable) )

}

imps2 = imps[["importance"]]

if (ncol(imps2) ==2){

imps2 = imps2[ClusterofInterest]

}

colnames(imps2) = model_label

imps2$Variable = rownames(imps2)

Imp_df<-merge(Imp_df, imps2,by = "Variable", all = TRUE )

}

newdf<-Imp_df[order(Imp_df$ImpairedProfile),]

newdf$ID <-seq.int(nrow(newdf))

#newdf$Variable<- VarTypes1[[2]][match(newdf$Variable, VarTypes1[[1]])]

dfImp<-melt(newdf, id = c('ID', 'Variable') )

#Rename Variables

##Try highlighing top value

me.2 <- dfImp %>%

group_by(variable) %>% mutate(color = value %in% head(sort(value, decreasing = TRUE),10) )

p<- ggplot(me.2, aes(x = Variable, y = value)) +coord_flip()

p<- p + geom_segment(size = 2,aes(x = Variable, xend = Variable, y =0, yend = value, color = color )) + scale_x_discrete(limits = newdf$Variable)

p<-p + facet_wrap(~variable, nrow=1) +theme(legend.position = "none", strip.text.y = element_text(angle=45), axis.title.x = element_blank(), axis.title.y = element_blank())

p<- p + geom_point() +ylab('Variable Importance') + ggtitle('Random Forest Variable Importance') +scale_color_manual(values = c("lightgrey", "black"))

plot(p)

```

### ROC Plots

```{r Women_RF_ROC, warning = FALSE, echo=FALSE, include=TRUE}

##Plot ROC

for (c in 1:length(CIs)){

CI<-CIs[c]

#Plot ROCs

model <- MR[[CI]]$FIT_RandomForest

Pred_RF <-predict(model, MR[[CI]]$DataSubset, type = "prob")

ROC_obj<-roc(response = MR[[CI]]$DataSubset$Cluster, predictor = Pred_RF[,CI], ci = TRUE)

ci.sp.obj <- ci.sp(ROC_obj, sensitivities=seq(0, 1, .01))

plot(ROC_obj)

plot(ci.sp.obj, type="shape", col="lightblue")

title(CI, line = 2)

print(model)

print(model$finalModel)

}

remove('dfImp', 'Imp_df', 'imps', 'imps2', 'me.2', 'model', 'model_RF', 'modelData_imputed', 'MR', 'NewData', 'newdf', 'Pred_RF', 'RF', 'ROC_obj', 'all_f', 'c', 'CI', 'CIs', 'groups', 'MatchingVars', 'model_label', 'namedVector', 'pvalscript', 'removed_30pmissing', 'removed_lowVar', 'removed_onelevelfactors', 'p' )

```

<div class="tocify-extend-page" data-unique="tocify-extend-page" style="height: 0;"></div>

# Version Info

```{r PrintAndSave, warning = FALSE, echo=TRUE, include=FALSE,cache=TRUE, fig.width = 10, fig.height = 18}

filename<- paste0("SexDiffEnvirSOM3to10clust_", format(Sys.time(), "%Y-%m-%d %I-%p"), ".RData")

save.image(file = filename)

print(paste("For future reference, the analyzed data shown here has been saved as", filename))

print(sessionInfo())

```
